# Development of Heterocyclic PPAR Ligands for Potential Therapeutic Applications

**DOI:** 10.3390/pharmaceutics14102139

**Published:** 2022-10-08

**Authors:** Sharma Arvind Virendra, Ankur Kumar, Pooja A. Chawla, Narsimha Mamidi

**Affiliations:** 1 Department of Pharmaceutical Chemistry, ISF College of Pharmacy, Moga 142001, Punjab, India; 2 Department of Chemistry and Nanotechnology, School of Engineering and Sciences, Tecnologico de Monterrey, Ave. Eugenio Garza Sada 2501, Monterrey 64849, Nuevo Leon, Mexico

**Keywords:** peroxisome proliferator-activated receptors, ligands, a thiazolidinedione, structure–activity relationship interactions

## Abstract

The family of nuclear peroxisome proliferator-activated receptors (PPARα, PPARβ/δ, and PPARγ) is a set of ligand-activated transcription factors that regulate different functions in the body. Whereas activation of PPARα is known to reduce the levels of circulating triglycerides and regulate energy homeostasis, the activation of PPARγ brings about insulin sensitization and increases the metabolism of glucose. On the other hand, PPARβ when activated increases the metabolism of fatty acids. Further, these PPARs have been claimed to be utilized in various metabolic, neurological, and inflammatory diseases, neurodegenerative disorders, fertility or reproduction, pain, and obesity. A series of different heterocyclic scaffolds have been synthesized and evaluated for their ability to act as PPAR agonists. This review is a compilation of efforts on the part of medicinal chemists around the world to find novel compounds that may act as PPAR ligands along with patents in regards to PPAR ligands. The structure–activity relationship, as well as docking studies, have been documented to better understand the mechanistic investigations of various compounds, which will eventually aid in the design and development of new PPAR ligands. From the results of the structural activity relationship through the pharmacological and in silico evaluation the potency of heterocycles as PPAR ligands can be described in terms of their hydrogen bonding, hydrophobic interactions, and other interactions with PPAR.

## 1. Introduction

Nuclear receptors are a type of protein that recognize steroid and thyroid hormones in the body [1]. Nuclear receptors tend to influence the growth, homeostasis, and metabolism of organisms by binding to DNA and regulating the expression of specific genes. As a result of their ability to modulate transcription, these are known as transcription factors [2,3]. The nuclear receptor superfamily’s peroxisome proliferator-activated receptor (PPAR) is a ligand-dependent transcription factor [4]. To build a heterodimer, all PPARs interact with the retinoid X receptor [5,6,7]. In 1990, PPARs were first observed in rodents [8]. PPARs are receptors associated with a superfamily of nuclear receptors that also contain steroids, retinoid receptors [9], thyroid hormone receptors, and Vit. D receptors [10]. They are multiple and persuasive regulators of various cellular functions and metabolic functions such as glucose and lipid homeostasis, cholesterol, and energy balance [11,12]. Peroxisome proliferator-activated receptors are a subgroup of the ligand-dependent transcription factor that contains peroxisome proliferator response elements as transcription factors to regulate transcriptional activity [13,14,15].

## 2. Structure of PPAR

**Figure 1:**
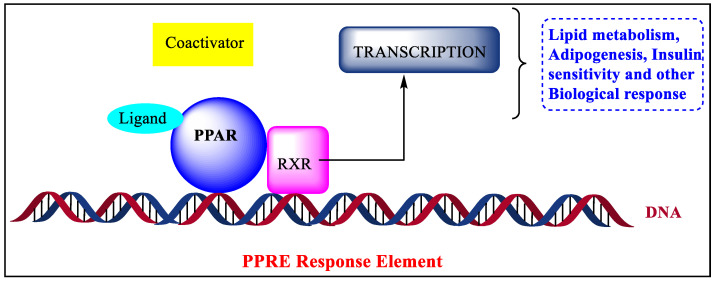
Mechanism of biological responses through PPAR.

**Figure 2:**
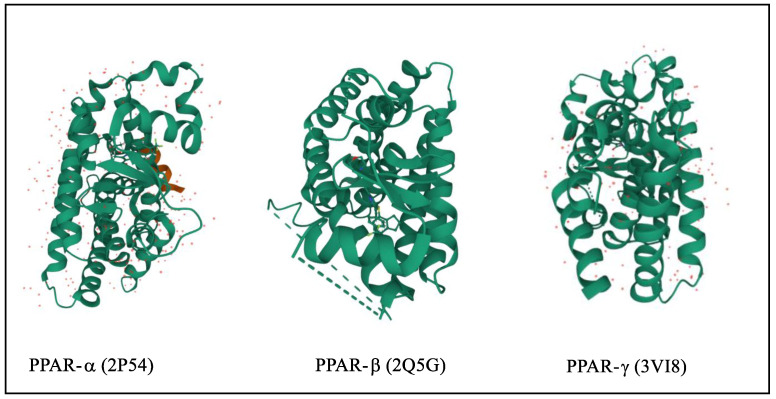
3D structure of PPARα/β/γ.

The three-dimensional structure of PPARs includes the N-terminus and C-terminus where DNA binding domain and ligand-binding domain are attached, respectively, [16]. They are translocated to the nucleus and heterodimerized with retinoid X receptors (RXR) [17]. In targeted genes, the PPREs (peroxisome proliferator hormone response elements) are particular DNA areas that interact with PPARs [18,19]. They are connate in the fatty acid-binding protein like PPAR-responsive gene promoters that activate transcription of multiple genes involved in various physiological processes shown in Figure 1 [20,21]. The protein structure of various PPARs is fetched from the protein data bank using three PDB ids such as 2P54 (PPARα), 2A5G (PPA, Rβ,), and 3VI8 (PPARγ), shown in Figure 2 [22,23,24,25,26].

## 3. Types and Expressions of PPARs

**Figure 3:**
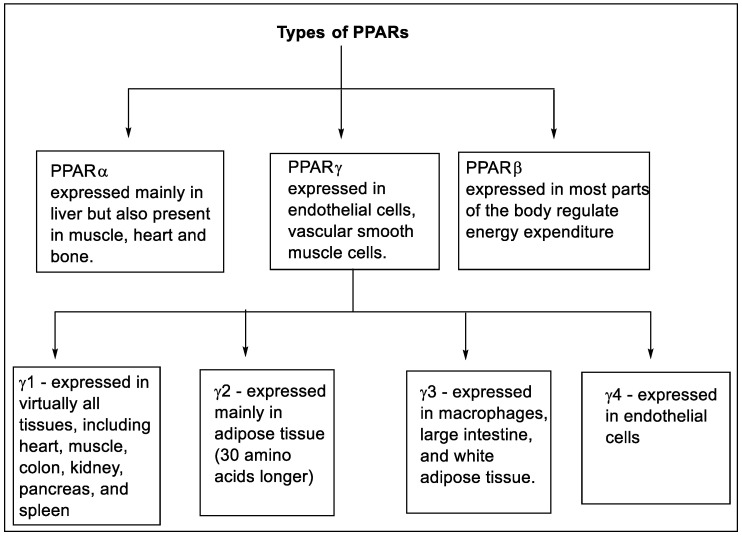
Types and expression of PPARs.

The transcriptional factors of peroxisome proliferator-activated receptors (PPARs) contain three different isoforms, namely nuclear receptor subfamily PPARα or NR1C1, PPARβ/δ or NR1C2, and PPARγ or NR1C3 [27,28,29]. These isoforms are expressed in multiple tissue and organs with identical character and ligand specificity [30]. The PPAR-α is expressed mainly in the liver but is also present in muscle, bone, and heart. The PPAR-δ is expressed in most parts of the body and regulates energy expenditure [31,32]. The PPAR-γ is expressed in vascular smooth muscle cells, and endothelial cells [33,34]. The expression of various types of PPARs is shown in Figure 3 [35,36,37].

## 4. Functions of PPARs

**Figure 4:**
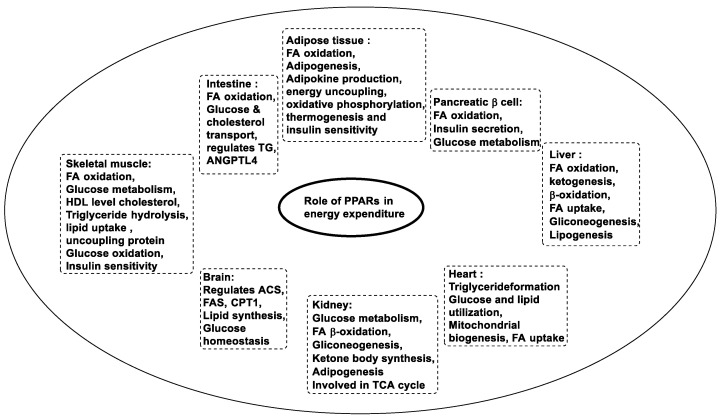
Functions of PPARs.

In the human body, the three forms of PPARs are responsible for the activation of most of the enzymes that are required for fatty acid oxidation, glucose metabolism, and lipid metabolism [38]. These PPARs perform specific or distinct functions such as cellular energy balancing, and maintenance of energy homeostasis on their own or by helping each other [39]. Energy metabolism at a molecular level through PPARs is still not explained but when ligands are attached to these receptors, they transcript the genes involved in regulatory energy functions [40]. The PPARα is involved in the activation of gene-encoding enzymes such as carnitine palmityl transferase 1 (CPT1), fatty acid transport protein, acyl-CoA dehydrogenase or oxidase, or synthase required in the fatty acid oxidation pathway [41]. It also reduces plasma triglyceride levels and increases high-density lipoprotein, which gives benefits such as ketogenesis [42]. The PPARβ controls fatty acid metabolism, increases insulin sensitivity, obesity resistance, suppression of macrophage-derived inflammation, and also the formation of oxidative muscle fibers. The PPARγ promotes fatty acid uptake, and adipokine production raises insulin sensitivity, also in adipogenesis [43,44,45,46]. Other functions associated with PPARs are described in Figure 4 [47,48,49].

In several areas of medicinal chemistry, heterocyclic molecules are indispensable [50,51]. These compounds constitute nitrogen, sulfur, and oxygen atoms with various positional combinations in the cyclic ring [52]. Medicinal chemists and researchers always explore heterocycles due to their vital contribution to drug discovery and design by boosting the novel lead moieties with potential biological action in medicinal chemistry [53]. Based on statistics, more than 85% of biologically potential chemical moieties contain heterocyclic motifs [54]. Some of the most common heterocycles are pyrimidine, imidazole, oxazole, tetrazole, triazine, triazle, thiazole, indole, pyridine, thiophene, pyrazole, coumarin, oxindole, furan, etc. [55,56,57,58]. These heterocyclic nuclei possess a broad spectrum of medicinal potential such as an anti-microbial [59], antimalarial [60], anti-anxiety [61], anti-cancer [62], anti-depressant [63], anti-tubercular [64], anti-virus [65], anti-protozoal [66], and anti-convulsant, etc. Several heterocyclic analogs have been reported to affect PPAR in different diseases [67,68,69,70,71,72]. Apart from commercially available marketed drugs, Table 1. depicts the patented drugs acting on PPARs.

In this review, we have tried to compile the literature of the recent past (2016–2022) discussing heterocyclic derivatives, wherein we have discussed various scaffolds showing their potential as PPAR ligands concerning different activities. The SAR of these derivatives will be discussed to pave the way for the development of new, safe, and economical ligands shortly. Various heterocyclic moieties that act on the PPAR receptors have been summarized and displayed in Table 2.

## 5. Recent Developments in the Medicinal Chemistry of PPARs

### 5.1. Thiazolidinediones

**Figure 5:**
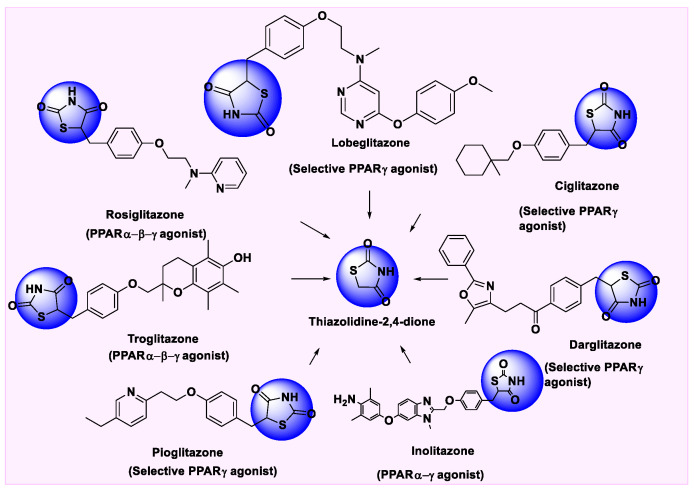
TZD scaffold containing PPAR ligands present in the market.

Sulfur and nitrogen-containing five-membered ring heterocycle is a thiazole, its non-aromatic simple being thiazolidine. At the point when thiazolidine is enriched further with two carbonyl groups at positions two and four, the ring framework is named 2,4-thiazolidinedione (TZD). TZD’s anti-diabetic properties were found in the early 1980s. Therefore, they were introduced during the 1990s as insulin-sensitizing glucose-lowering drugs for the treatment of type 2 diabetes mellitus (T2DM). They work fundamentally by initiating PPAR-γ, which thus controls glucose, lipids, and protein digestion. Troglitazone was the first medicine containing TZD pharmacophore but it had to be removed in the United States because of idiosyncratic hepatotoxicity that resulted in fatalities. Based on reports from other nations, it was also discontinued in the United Kingdom. In the years 1999 and 2000, rosiglitazone and pioglitazone were introduced in the US and Europe, in combination with other hypoglycaemic agents such as, e.g., thiazolidinedione plus metformin [128]. Several existing drugs that have a TZD motif in their structure have been displayed in Figure 5. Many chemists have reported the information on structurally modified TZD molecules exhibiting potential towards PPARs receptors that has been been described here.

**Figure 6:**
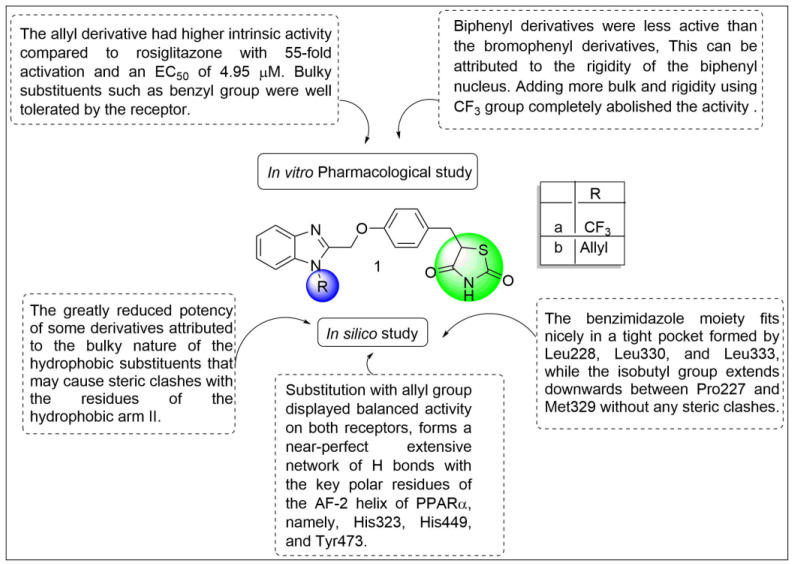
Structural activity relationship of thiazolidinediones hybrids (**1**) as potent PPAR ligands.

Darwish et al. designed and synthesized some thiazolidinedione derivatives with the aim of finding agonistic activity at the PPARγ receptor. According to the study, nineteen derivatives of thiazolidinedione were synthesized in which nine compounds exhibited promising activity. All the derivatives were tested for PPARγ receptor agonistic activity by using a luciferase-based genetic reporter assay. The result showed that bromophenyl and biphenyl-containing compounds exhibited moderate activity whereas five compounds showed five-fold more activity. There was a rigidity of the biphenyl nucleus, which was why bromophenyl derivatives were more active than biphenyl derivatives. As shown in Figure 6, trifluoro methyl (**1a**) containing derivatives diminishes activity due to their bulk and rigidity. The allyl derivative (**1b**) exhibited higher agonistic activity compared to rosiglitazone with 55-fold activation and an impressive EC_50_ of 4.95 μM in the human PPAR-γ transactivation assay [83].

**Figure 7:**
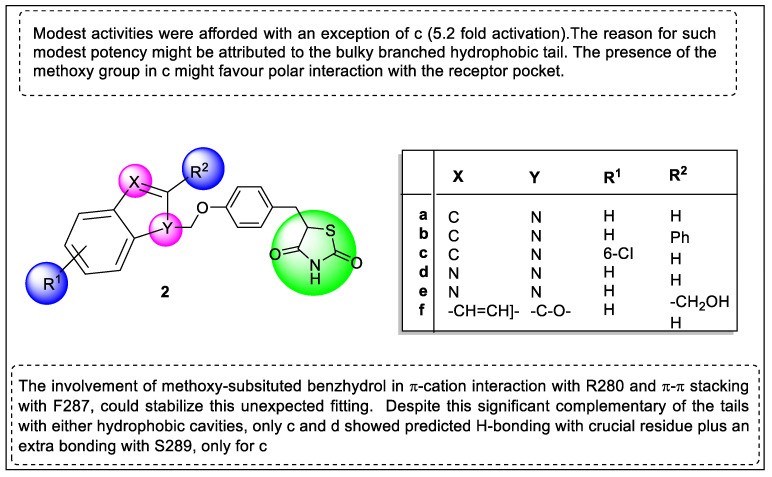
Structural activity relationship of thiazolidinedione hybrids (**2**) as potent PPAR ligands.

The same group of authors continued their research on PPAR-γ receptors and designed benzhydrol and an indol-based series of derivatives in the search for new candidates of thiazolidinediones chemotypes as dual PPAR-γ/FFAR1 agonists. The optimization of compounds based on the exhibiting affinities towards PPAR-γ and the FFAR1 receptor at the low micromolar range selected compounds **2a**, **2c,** and **2d**. The in vivo pharmacological study resulted in the compound **2c** (EC_50_ = 17.75 µM) exhibiting better anti-hyperglycemic activities than **2a** (21.34 µM), **2c** (Inactive), and the reference compound (Rosiglitazone) in fat-fed animal models. Molecular docking studies were carried out to check the binding with the receptor. The SAR study of synthesized series and their important substitution is shown in Figure 7 [129].

**Figure 8:**
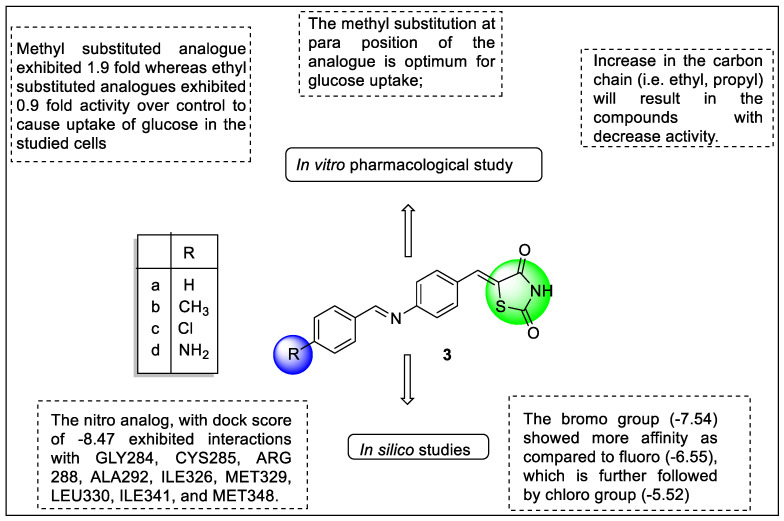
Structural activity relationship of Thiazolidinediones hybrids (**3**) as potent PPAR ligands.

Chhajed et al. designed and synthesized a benzylidene amino-benzylidene-thiazolidine-2,4-dione scaffold containing derivatives to find novel PPAR-γ agonists. The research framework began with virtual screening by using molecular docking studies on the ligand-binding domain of PPAR-γ that filtered out ten compounds based on their affinity. Therefore, these compounds were processed for synthesis. The molecular docking was carried out on the 3-D structure of PPAR-γ (PDB ID: 2XKW) using a molecular design suite (MDS). From the molecular docking studies, the researchers observed significant binding of the derivatives with the receptor having a good docking score wherein the substitution at a para position on phenyl ring possessed better affinity comparison with the ortho- and meta-substituted compounds. Further, the substitution of amino and nitro at the para position showed a great affinity with score of −9.22 and −8.47, respectively. The binding of TZD molecules was demonstrated via H-bonding, where aniline, carbonyl oxygen, and benzylidene moiety bound with Arg288, Ile326, and Ile281 or Ile341, respectively. The authors also noted H-bonding and pi-interaction with Ala292 and Met329. Gly284, Leu330, Cys285, and Met348. Synthesized molecules were tested for glucose uptake assay for the 3T3-L6 cell line using rosiglitazone as a standard reference. The results revealed that compounds **3a**, **3b**, **3c,** and **3d** increased glucose uptake 1.6, 1.8, 1.6, and 1.7 times more than reference compounds, respectively. The study showed that compounds bearing electron-donating groups such as NH_2_ and CH_3_ and electron-withdrawing groups such as the halogens (Br, F, and Cl) were responsible for PPAR-γ agonist activity. Lipophilicity also played a significant role in the activity, which was corroborated by the introduction of methyl and ethyl groups to the phenyl ring. The results of in vitro pharmacological activity matched with the docking study (Figure 8) [84].

**Figure 9:**
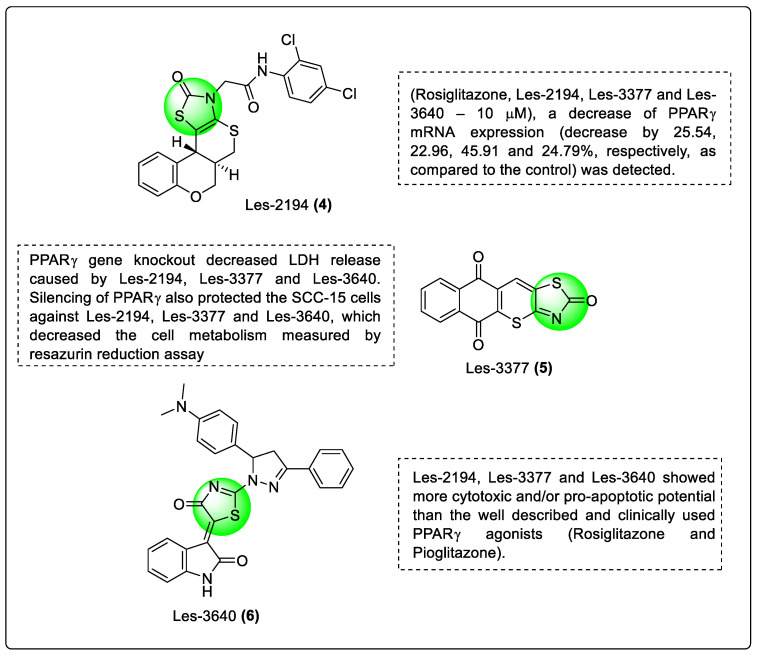
Structural activity relationship of thiazolidinedione (**4**, **5**, and **6**) hybrids as potent PPAR ligands.

Szychowski and co-workers reported some 4-thiazolidinedione derivatives as new candidates for the treatment of cancer. In this study Les-2194 (**4**), Les-3377 (**5**), Les-3640 (**6**) (4-thiazolidinedione derivatives) along with rosiglitazone, and pioglitazone were tested for their involvement in PPARs receptors in the mechanism of pro-apoptotic action and their cytotoxicity using the squamous carcinoma (SCC-15) cell line. The PPARα, PPARβ, and PPARγ mRNA expression were examined using 4-thiazolidinone compounds and PPAR-specific siRNA. Cell survival, cell metabolism, and caspase-3 activity were also assessed after PPARα, PPARβ, and PPARγ siRNA gene suppression. The mRNA expression of PPARs decreased in SCC-15 cells treated with Les-2194, Les-3377, and Les-3640. The cytotoxic effect of the examined drugs (50 µM) was prevented by PPARγ knockdown. In SCC-15 cells, it was found that the new anticancer 4-thiazolidinone compounds act mostly via the PPARγ pathway (Figure 9). The authors found that all the compounds emerged as PPARγ modulators. Silencing the PPARγ gene raises the PPARα and PPARγ mRNA F in SCC-15 cells, which is interesting. The anticancer activity of the newly discovered chemicals was better than that of rosiglitazone and pioglitazone [130].

**Figure 10:**
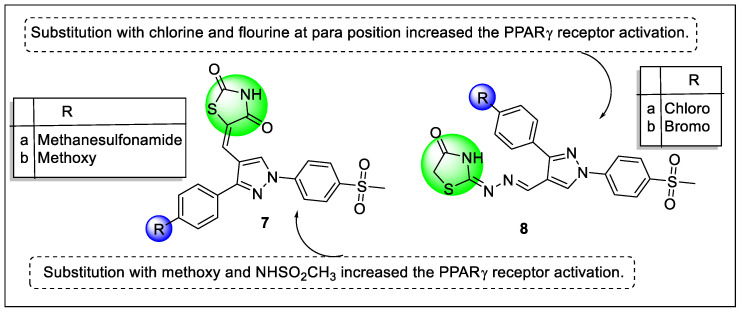
Structural activity relationship of thiazolidinedione (**7** and **8**) hybrids as potent PPAR ligands.

Abdellatif et al. designed and synthesized pyrazole containing two series of thiazolidine and thiazolidinone derivatives attached with methylene or methylenehydrazone linkers to target the PPARγ receptor. The luciferase-based genetic reporter assay carried out for all synthesized analogs to screen the activity of PPARγ receptors that showed methanesulfonamide (**7a**) and methoxy (**7b**)-substituted thiazolidinedione derivative along with chloro (**8a**) and bromo (**8b**)**-**substituted thiazolidinone derivatives increased the PPARγ receptor activation and displayed 52.11%, 59.63%, 63.15%, and 55.24% PPAR-γ transactivation, respectively. The docking study revealed that the Y shape was successfully adopted by the tested compounds **7a**, **7b**, **8a,** and **8b**, and two of them, **7a** and **8b**, docked with PPAR-γ (PDB ID: 4O8F) in the same region as rosiglitazone, while the other two interacted with the receptor in the left motif as pioglitazone (Figure 10) [85].

**Figure 11:**
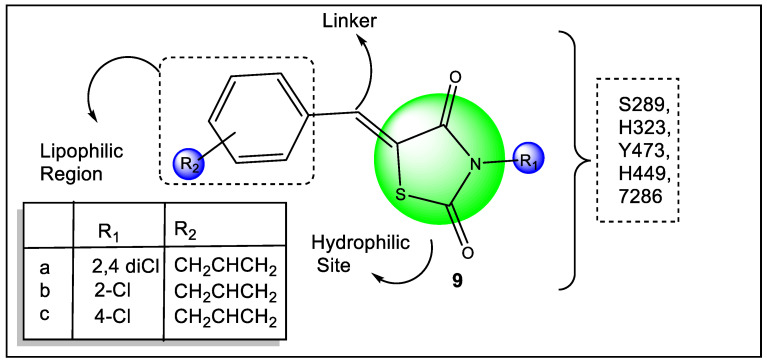
Regions of thiazolidinedione (**9**) hybrids are required for binding to PPAR.

Srivastava et al. reported 2,4-thiazolidinedione derivatives (Figure 11) and screened for anti-diabetic, free radical scavenging activities, and anti-inflammatory activity. The *in-silico* studies were also carried out using molecular docking to analyze some important structural features in the binding manner of reference anti-diabetic drugs with the active protein binding sites of PPARγ (PDB ID: 2PRG). Compound **9a** (dichloro derivative) exhibited significant antidiabetic potential along with anti-inflammatory and antioxidant potential. Numerous possible interactions were analyzed and compared with the reference compound (pioglitazone). Compounds **9a**, **9b**, and **9c** had docking scores of −11.6930, −10.1553, and −11.1008, respectively, which were quite close to pioglitazone (−12.8116). The amino acid residues Ser A289 and His A323 demonstrated two hydrogen bond interactions with compound **9a**. Compound **9b** had one keto group that hydrogen-bonded with water in combination with Ser 342 and another keto group that hydrogen-bonded with another molecule of water in interaction with Arg. As seen in Figure 9, compound **9c** establishes a hydrogen bond with Ser A342. By forming a hydrogen bond with Glu A343 and another interaction with HOH 604 linked with Ser 342, pioglitazone demonstrated a binding pattern [86].

**Figure 12:**
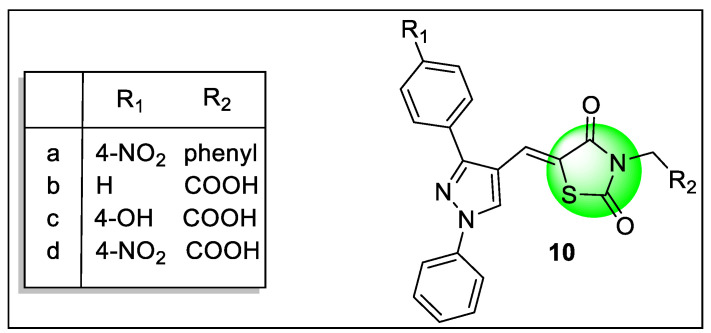
Series of thiazolidinedione hybrids (**10**) as potent PPAR ligands.

Bansal et al. also reported TZD derivatives (Figure 12) associated with the pyrazole motif, docked against PPAR-γ and α-amylase, and further screened for antidiabetic, antioxidant, and anti-inflammatory activities. Compound **10d** was found to have a strong blood glucose reducing effect and was a promising inhibitor of α-amylase. Compound **10a** showed it had potential as an anti-inflammatory drug that reduced MDA, IL-, and TNF- (inflammatory markers), as well as having some antioxidant activity. Compounds **10b** (−15.13), **10c** (−16.79), and **10d** (−17.44) had higher docking scores in the PPARγ interaction than the pioglitazone (−12.605). Hydrogen bond interactions were observed in compound **10b** which included the interaction of one keto group with an HOH molecule along with Arg288, another keto group with Ser342, and an acidic hydroxyl group with an HOH molecule in association with Ser342. The hydroxyl group of compound **10c** showed H-bonding interactions with HOH when combined with Glu272; additionally, other H-bonding interactions involving Glu272, and Gln271 were found. In association with Gly284; compound **10d** revealed only one water molecule binding. The acidic head of pioglitazone displayed interaction with the HOH molecule associated with Arg288 The structure of potent derivatives is shown in Figure 12 [131].

**Figure 13:**
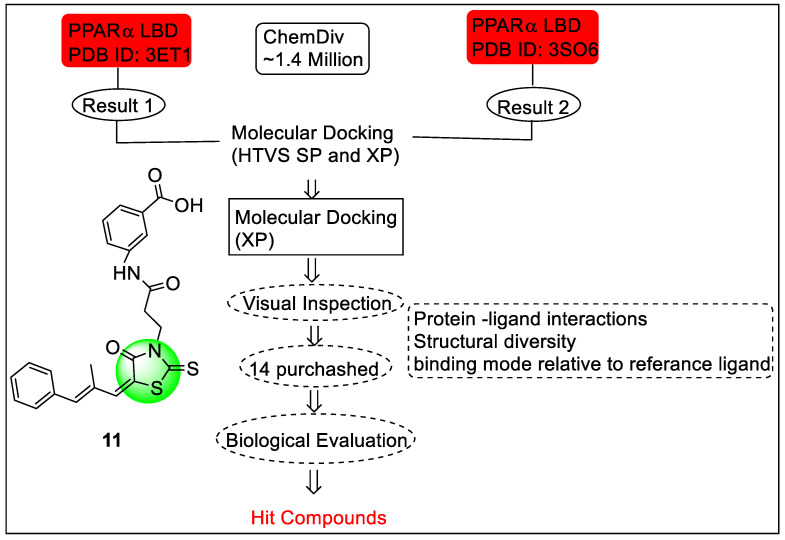
Workflow for the virtual screening of thiazolidinedione hybrids (**11**) as potent PPAR ligands.

Dai and co-workers carried out the virtual screening and biological evaluation by analyzing the change in gene profile in the humanized mice treated for the discovery of a novel potent selective PPARα agonist. Virtual screening was performed on the crystal structure of the PPARα and two different PDB IDs, 3ETI and 3SP6, were selected (Figure 13). Molecular docking was also performed by using Glide 5.5 in Schrödinger2009. Based on the study, compound **11** was found as a potent PPARα agonist where it regulated the target genes responsible for functions such as fatty acid metabolism, activities related to lipid-lowering, and inflammation. The structure of potent compound **11** is shown in Figure 11. [87].

**Figure 14:**
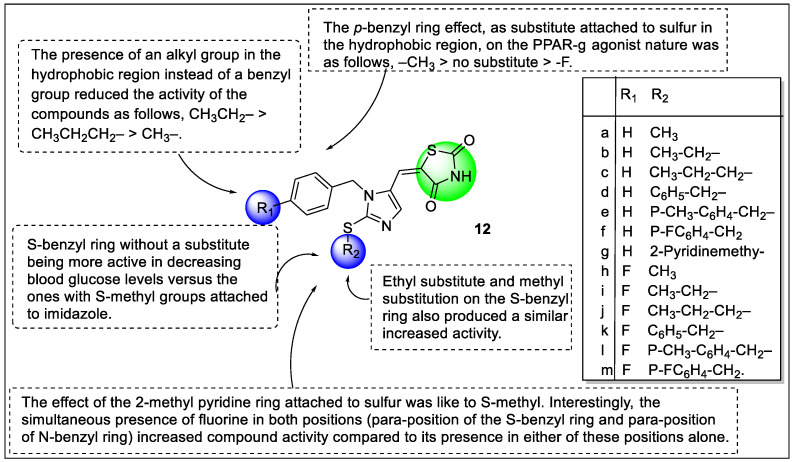
Structural activity relationship of thiazolidinedione hybrids (**12**) as potent PPAR ligands.

Recently, Shakour et al. reported a new series of imidazolyl-methyl-1-2,4-thiazolidinediones **12** (**a**–**m**) along with in silico studies. Anti-hyperglycemic activity was carried out on 3T3 cells compared with a diabetic control group. Molecular docking was performed on the PPARγ receptor using PDB code 5Y2O with a considerable RMSD value (0.02). All synthesized compounds were docked with the help of MOE software. Based on the results of in silico studies, these synthesized compounds exhibited a good score ranging from −10.55 to −12.69 kcal/mol compared with pioglitazone (−11.17 kcal/mol). Compounds with methyl benzene substitution at R_2_ position and fluoro at R_1_ and ethyl substitution at R_2_ showed cation-π bond interaction and hydrogen bond with Arg288 and His449, respectively. Moreover, it was demonstrated that fluoro-substituted analog reduced 0.09-fold the expression gene of PPARγ in cultured adipocytes compared to the control group. The study described the SAR of imidazolyl-methyl-1-2,4-thiazolidinedione derivative as those compounds containing non-substituted S-benzyl ring were comparatively more active than imidazole containing S-methyl groups in decreasing blood glucose levels. Substitutions at the hydrophobic region affect the range of the PPARγ agonistic activity such as methyl group substitution at sulfur having more potency than non-substituted and fluorine substitution at sulfur. Moreover, the benzyl group replaced with the alkyl group at the hydrophobic region reduced the activity as follows: CH_3_CH_2_– *>* CH_3_CH_2_CH_2_– *>* CH_3_– as shown in (Figure 14). Although the literature has reported that PPARγ agonists are associated with an increase in body weight, interestingly in this study **12e** did not induce variation in body weight as compared to pioglitazone [88].

### 5.2. Oxazole and Oxadiazole

Oxazole is a five-membered heterocyclic ring compound that contains oxygen and nitrogen at the first and third positions, respectively, and also one carbon situated between oxygen and nitrogen.

**Figure 15:**
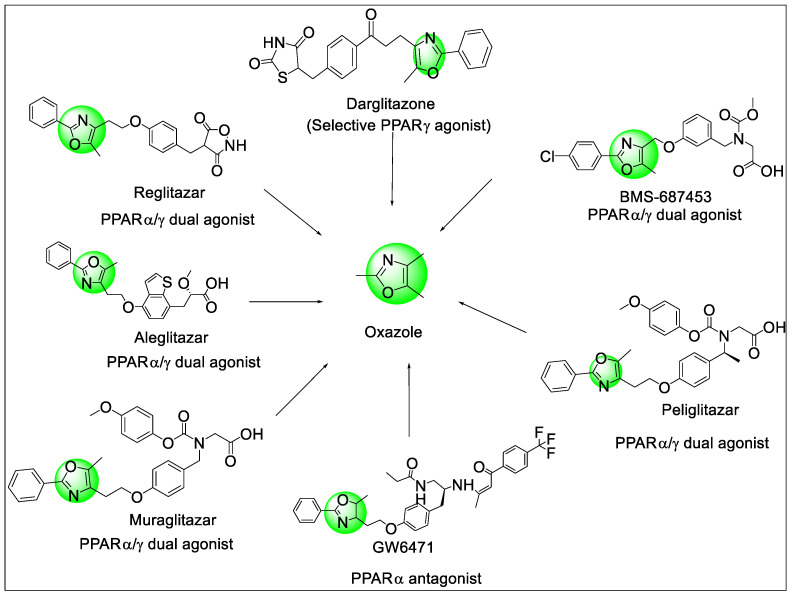
Oxazole scaffold containing PPAR ligands present in the market.

Oxadiazoles are five atoms containing heterocycles with two nitrogens and one oxygen. These atoms can have a different distribution in the ring to generate 1,2,4-oxadiazole, 1,3,4-oxadiazole, 1,2,5-oxadiazole, or 1,2,3-oxadiazole compounds. The background of oxazole-containing compounds suggests that they exhibit promising potential as PPAR ligands and also generate multiple side effects. In July 2013, aleglitazar was stopped in phase III due to heart failure, bone fractures, and gastrointestinal bleeding; muraglitazar was suspended in phase III due to risks regarding cardiovascular events; ragaglitazar was withdrawn during phase III because of tumors found in the urinary bladders of mice (Figure 15). The chemical and biological potential of oxazole analogs as PPAR ligands have been studied widely over the years.

**Figure 16:**
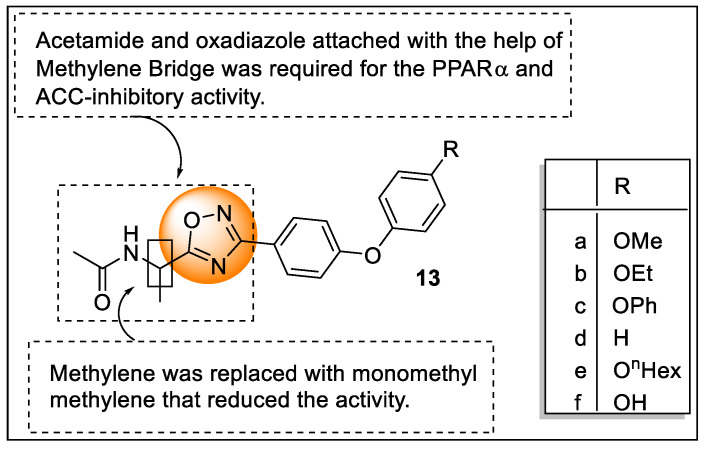
Structural activity relationship of oxadiazole hybrids (**13**) as potent PPAR ligands.

For the treatment of various metabolic disorders, targeting PPARs is a well-established strategy, along with inhibiting Acetyl-CoA carboxylase (ACCs) gaining attention via the prevention of de novo lipogenesis. Therefore, Okazaki et al. synthesized a series of acetamides as ACC2 inhibitors along with the dual agonistic activity of PPARα/δ based on the structural or molecular similarity between these targets. These synthesized analogs were evaluated for their ACC-inhibitory activity and PPAR-agonistic activity which revealed analogs **13f** exhibited significant activity. The structural activity relationship study displayed that analogs **13a**, **13b**, and **13d** were active at a submicromolar concentration where **13b** and **13d** exhibited potency toward ACC2 and PPARα/γ. The phenoxy group (**13c**) containing PPAR-selective analogs (**13f** and **13a**) also showed potency towards ACC inhibition. Acetamide and oxadiazole attached with the help of a methylene bridge were required for the PPARα- and ACC-inhibitory activity and the replacement of methylene with monomethyl methylene reduced the activity (Figure 16). The enantiomer of most potent analogs **13c** and **13d** were synthesized and the researcher found that the (S)-isomers of **13c** and **13d** displayed more ACC2-inhibitory activity than the (R)-isomer [89].

**Figure 17:**
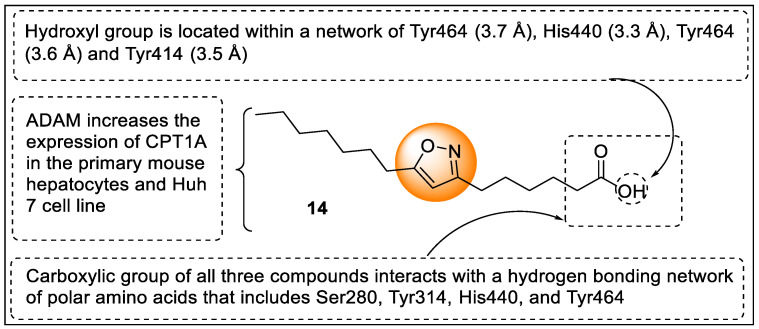
Structural activity relationship of oxazole hybrids (**14**) as potent PPAR ligands.

Arnesen and coworkers identified two oxohexadecenoic acids isolated as isomeric forms from the marine microalga *Chaetoceros karianus.* The IUPAC names of these acids are oxohexadecenoic acid (7*E*).-9-oxohexadec-7-enoic (OFAI), and (10E)-9 -oxohexadec-10-enoic acid (OFAII), and they exhibit the dual agonistic effect of PPARα/γ. Based on the potency of these acids towards PPARα/γ, the authors designed an analog of 3,5-disubstituted isoxazole fatty acid to find a selective agonist of PPARα. The molecular modeling of synthesized analog 6-(5-heptyl-1,2-oxazol-3-yl)hexanoic acid (ADAM) displayed binding with Tyr501, His447, and Ser317 amino acids required for PPARα activation. Moreover, they had trouble attaching with the polar part of amino acid residues in PPARγ that is responsible for the AF2 stabilization and co-activator recruitment (Figure 17). The pharmacological evaluation revealed that ADAM increases the expression of CPT1A in the primary mouse hepatocytes and Huh 7 cell line [90].

**Figure 18:**
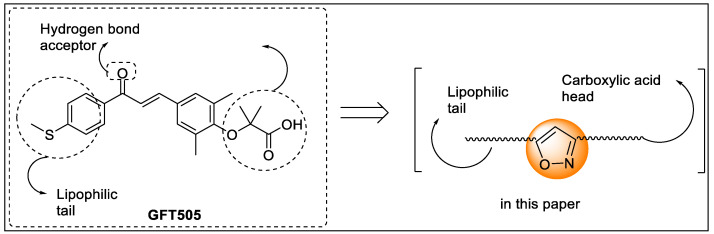
The rationale behind choosing oxazole hybrids as potent PPAR ligands.

**Figure 19:**
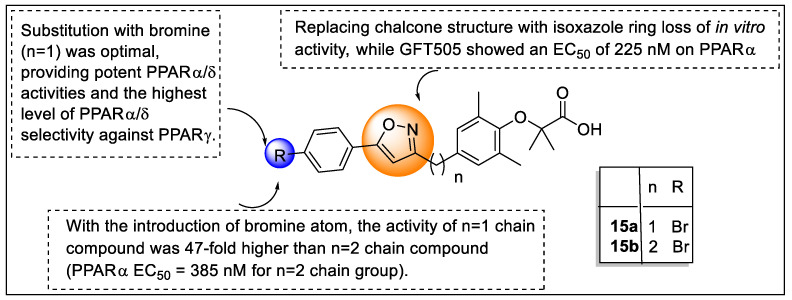
Structural activity relationship of oxazole hybrids (**15**) as potent PPAR ligands.

Jiang and their team reported a new series of isoxazole analogs as a selective agonist of PPARα/δ receptors (Figure 18). These synthesized analogs were screened using nuclear hormone assay (NHR assay) where GW7647, L−165,041, and troglitazone were used as a standard for PPAR α, δ, and γ, respectively, revealing that extension of the carbon chain diminishes activity. For example, chalcone replaced with isoxazole ring led to a loss of in vitro activity. Analogs **15a** and **15b** possessed 8–28 fold more PPARα agonistic activity along with good EC_50_ values of 8 nM and 27 nM, respectively. However, cyclization of the isoxazole and phenyl groups (polycyclic isoxazole) showed negligible potential in improvisation of activities. Among all of the synthesized compounds, analog **15a** exhibited potency and selectivity towards PPARα/δ receptors with PPARα/δ/γ EC_50_, EC_50_ γ/α ratio, and EC_50_ γ/δ ratio value was 8/5/2939 nM, 367, 588, respectively, (Figure 19) [91].

**Figure 20:**
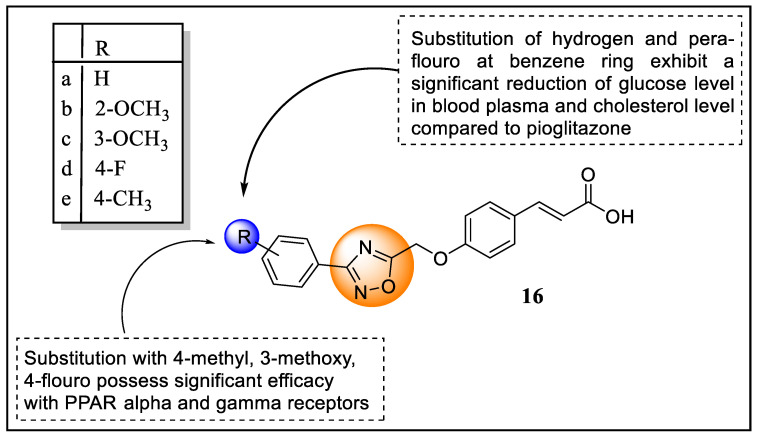
Structural activity relationship of oxadiazole hybrids (**16**) as potent PPAR ligands.

To discover new drug candidates for the treatment of type 2 diabetes that possess characteristics of both thiazolidinedione and fibrates, Kaur et al. designed new 1,2,4-oxadiazole-based trans-acrylic acid analogs as PPARα/γ dual agonists. These analogs had aryl or methylene linkers in between the lipophilic tail and pharmacophore head to serve as PPARα/γ dual agonists in silico studies of these analogs using autodock vina along with toxicity, and ADME properties were performed. The in-silico studies revealed that analogs substituted with 2-methoxy (**16b**), 3-methoxy (**16c**), 4-fluoro (**16d**), and 4-methyl (**16e**) groups displayed good results based on affinity scores compared with Pioglitazone on PPARγ. The lipophilicity (iLogP) range of analogs was found to be 0.92 to 3.19. Further, the in vitro pharmacological evaluation of PPARα/γ reported that three analogs (4-F (**16d**), 3-OCH_3_ (**16c**), and 4-CH_3_ (**16e**)) possessed significant efficacy with α and γ receptors (Figure 20). The in vivo pharmacological screening of the diabetic rat model revealed that non-substituted and fluoro-substituted analogs exhibited a significant reduction in glucose levels in blood plasma and cholesterol levels compared to pioglitazone [92].

### 5.3. Benzoimidazole

**Figure 21:**
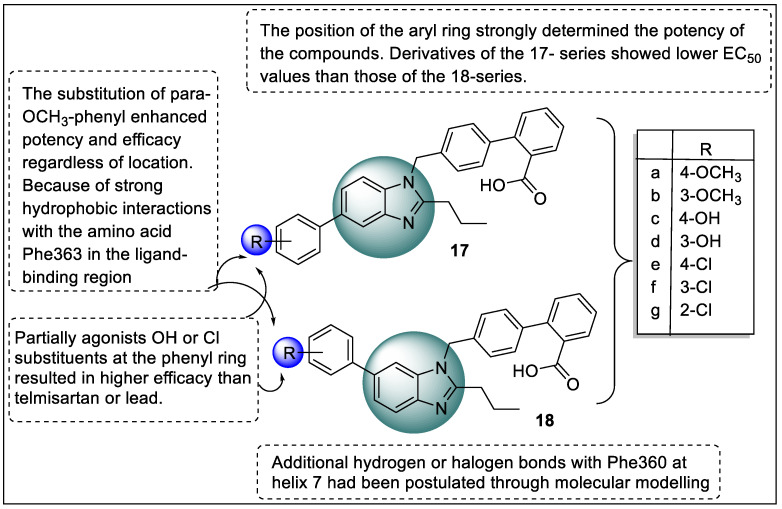
Structural activity relationship of Benzimidazole hybrids (**17 and 18**) as potent PPAR ligands.

At the fourth and fifth locations of the imidazole ring, the benzimidazole molecule is formed by combining the benzene and imidazole ring systems. They have both acidic and basic characteristics. The NH group is both acidic and basic in this case. Obermoser et al. synthesized 5- or 6-substituted benzo[d]imidazoles as PPARγ agonists. Therefore, the effect of aryl substituents at positions five and six on the pharmacological profile of the partial PPARγ agonist 4’-((2-propyl-1*H*-benzo[d]imidazol-1-yl)methyl)-[1,1’-biphenyl]-2-carboxylic acid was examined. The substitution of para-OCH_3_-phenyl enhanced potency and efficacy regardless of location. Due to the strong hydrophobic interactions with Phe363 in the ligand-binding region, both drugs are complete agonists. Partial agonist OH or Cl substitution at the phenyl ring resulted in higher efficacy than telmisartan or lead. Moreover, hydrogen or halogen bonds with Phe360 at helix 7 were postulated through molecular modeling (Figure 21). These interactions were thought to fix helix 7, and induced a partial agonist conformation of the protein receptor. The results of the luciferase transactivation correlate quite well with the theoretical concerns of those from a time-resolved fluorescence resonance energy test as well as those from a hPPARγ -LBD assay recruitment and corepressor recruitment (TR-FRET) experiment in which the coactivator (TRAP220, PGC-1a) and corepressor recruitment patterns of (NCoR1) release were studied [93].

**Figure 22:**
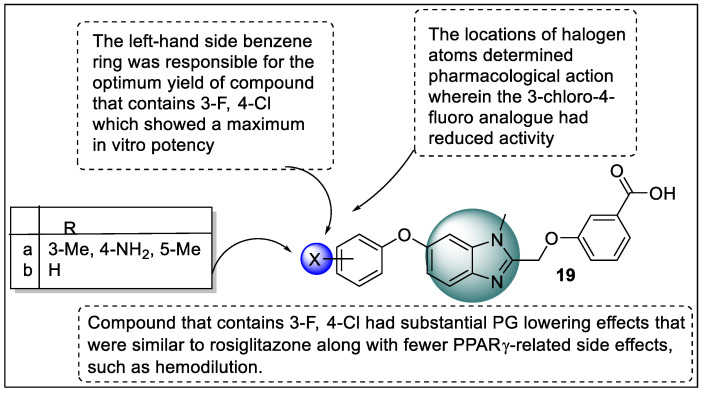
Structural activity relationship of Benzimidazole hybrids (**19**) as potent PPAR ligands.

Shinozuka and co-workers synthesized novel PPARγ ligands to avoid adverse effects related to PPARγ ligands via inhibiting with helix 12 ligand-binding site. The interaction with helix 12 is responsible for the effects of the TZD group of drugs so a selective PPARγ agonist (DS-6930) was identified as a lead compound. Subsequently, in vivo efficacy*,* in vivo ADME properties, and in vitro activity were performed. GAL4-PPAR-LBD reporter gene assays were used to test PPARγ transcriptional activities on COS-7 cells compared with rosiglitazone. The benzene ring in **19a** was responsible for the optimum yield of compound **19b** which showed a maximum in vitro activity. The locations of halogen atoms determined therapeutic action wherein the analog **19b** (3-Cl-4-F) had reduced activity. In ZDF rats, compound **19b** had substantial PG lowering effects that were the same as rosiglitazone along with fewer PPARγ-related side effects, such as hemodilution. The binding mechanism of compound **19b** with PPARγ was investigated and shown to have no contact of **19b** on Tyr473 at helix 12. The lipophilic interactions of the 4-chloro-3-fluorophenoxy group also found that compound **19b** produced minor hepatotoxicity during toxicological testing (Figure 22) [94].

**Figure 23:**
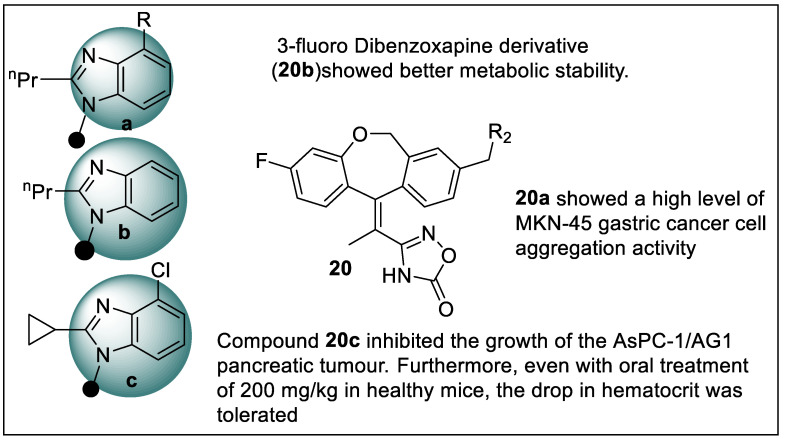
Structural activity relationship of Benzimidazole hybrids (**20**) as potent PPAR ligands.

Yamamoto et al. synthesized dibenzooxepine derivatives and pharmacological evaluations were performed. These compounds were tested using the following operation that includes Chimeric GAL4-PPARγ transactivation reporter assay (for PPARγ agonistic activity using transiently transfected HEK293EBNA cells), MKN45 cell aggregation assay, metabolic stability assay human liver microsomes, pharmacokinetic studies, xenograft study, RT-qPCR, and Hematocrit test. The summarized result of the biological evaluation showed that **20a** possessed a high level of MKN-45 gastric cancer cell aggregation activity, which was a sign of PPAR-induced cancer differentiation; moreover, **20a** was metabolically unstable during activation so Yamamoto et al. identified a metabolically weak area of the work and effectively discovered a 3-fluoro dibenzoxapine derivative (**20b**) with better metabolic stability. When compared to **20b**, imidazo[1,2-a]pyridine derivative (**20c**) showed powerful MKN-45 gastric cancer cell aggregation activity as well as superior PK profiles. In mice, compound **20c** inhibited the growth of the AsPC-1/AG1 pancreatic tumor (Figure 23). Furthermore, even with oral treatment of 200 mg/kg in healthy mice, the drop in hematocrit was tolerated [95].

**Figure 24:**
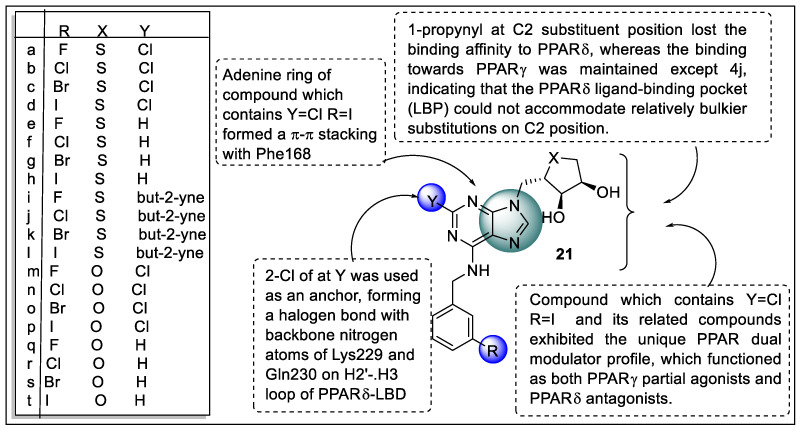
Structural activity relationship of Benzimidazole hybrids (**21**) as potent PPAR ligands.

An et al. discovered 1′-homologated adenosine derivatives **21a**–**21t** as modulators of dual PPARγ/δ with a lack of binding towards adenosine receptors that was possible due to the 1′-homologation strategy. The biological evaluation of these synthesized compounds was also performed to measure PPAR-binding profiles using the time-resolved fluorescence resonance energy transfer (TR-FRET)-based nuclear receptor-binding assay effect on adiponectin biosynthesis using the adipogenesis model of hBM-MSCs and pharmacological profile of the dual PPARγ/δ modulator using a TR-FRET assay. The results of the assay showed that none of the compounds showed binding towards PPARα but analogs **21a**, **21c, 21d,** and **21n**–**21p** (2-Cl derivative) showed promising binding towards the PPARγ/δ receptor. Especially, compound **21i**−**21l** with 1-propynyl at the C2 substituent position lost the binding affinity to PPARδ, whereas the binding towards PPARγ was maintained except 4j, indicating that the PPARδ ligand-binding pocket (LBP) could not accommodate relatively bulkier substitutions on the C2 position. In hBM-MSCs, analogs **21a, 21c, 21d, 21i, 21k, 21l, 21n, 21o**, and **21p** significantly increased adiponectin production compared to the IDX vehicle control during adipogenesis, but analogs **21e**–**21h** and **21q**–**21t** were inactive at PPARs. Analogs **21d** and **21p** emerged as the most powerful compounds in the 4′-thio- and 4′-oxonucleosides, respectively, whose adiponectin secretion-promoting PPAR/dual modulators may have therapeutic promise against cancer and metabolic illnesses, according to the biological screening (Figure 24) [96].

**Figure 25:**
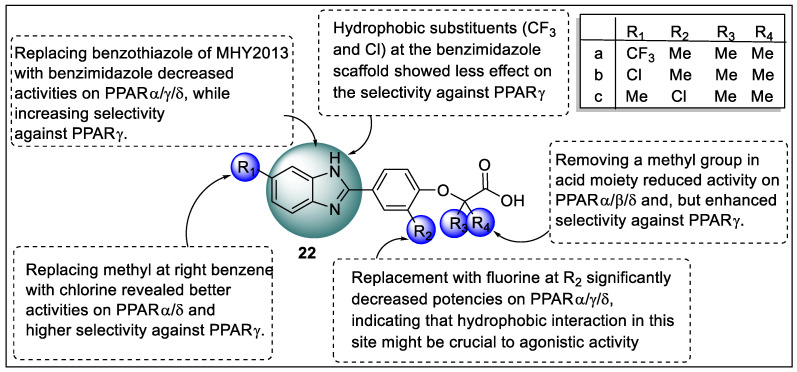
Structural activity relationship of Benzimidazole hybrids (**22**) as potent PPAR ligands.

Li and the group reported analogs of benzimidazole scaffold as a dual agonist of PPARα/δ for the treatment of non-alcoholic fatty liver disease. A cell-based assay was used for the evaluation of in vitro activities of analogs on PPARs. The results from the structural activity relationship of benzimidazole derivatives showed that compounds **22a** and **22b** exhibited a lower selectivity towards PPARs whereas they substituted additional CF_3_ and Cl in the benzimidazole motif. In particular, substituting chlorine where there was methyl at the right benzene yielded compound **22c**, which had stronger PPARα/δ activity and showed selectivity against PPARγ compared to the parent compound. Additionally, fluorine replacement greatly reduced agonistic activity on PPARα/δ/γ, demonstrating that hydrophobic interaction in this location is important for agonistic activity. Following that, the researcher looked at the significance of the carboxylic acid motif. Removing a methyl group from the acid moiety decreased PPARα/δ/γ activity, but increased selectivity against PPARγ. The docking study was also performed for the most promising candidate on PPARα/δ (PDB code: 3CI8 and 1GWX) using MOE. Compound **22c** displayed significant fitting toward PPARα/δ binding pockets. The H-bonding interaction of compound **22c** with His440, Tyr314, and Ser280 was due to the presence of carboxylic acid. Furthermore, the benzimidazole scaffold was placed into a PPARα hydrophobic pocket. The binding manner of compound **22c** toward PPARδ was different from PPARα. The reason behind this was the rotation angle between benzimidazole and benzene present in compound **22c**. The carboxylic acid of Compound 4 displayed three hydrogen bonds with His449, Tyr473, and His323. Furthermore, the oxygen atom of compounds **22c** and His449 showed hydrogen bonding interaction (Figure 25) [97].

**Figure 26:**
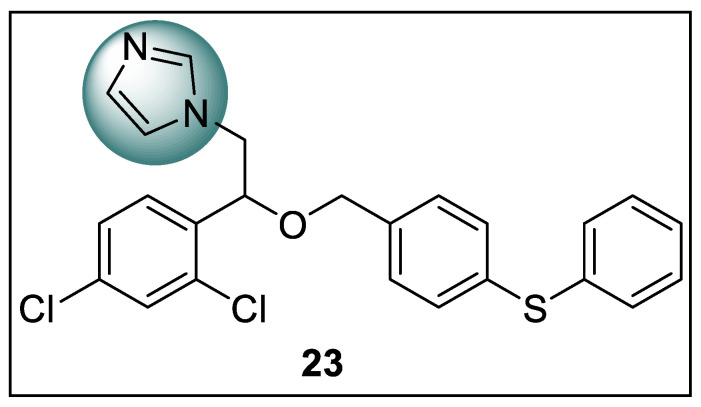
Structure of fenticonazole (**23**).

Since the use of PPARγ full agonists in the treatment of type-2 diabetes mellitus has been linked to several negative side effects, the development of new alternative candidates as PPARγ ligands has gotten a lot of attention in recent years. The old drug library was repurposed using a host-based medication repurposing technique using SB-VHTS. Herein Ma et al. identified fenticonazole (Figure 26), which was a CFDA-approved imidazole-containing antifungal drug, as a potent PPARγ modulator. The pharmacological investigation of fenticonazole (**23**) showed such desired drug properties primarily by potently activating the expressions of adiponectin and GLUT4, inhibiting the expressions of TNF-α, IL-6, and IL-1, blocking PPAR Ser273 phosphorylation that leads to adipogenesis without mediation by CDK5 and inducing the expressions of key adipogenic genes CD36, LPL, AP2, FASN, C/EBP, and PPARγ. Molecular docking validation was also performed at the active site of PPARγ (PDB code: 6MS7) using the CDOCKER mode in Discovery Studio 3.1, resulting in a promising binding mode between the fenticonazole and ligand-binding site of PPARγ, which included a hydrogen-bonding network between the oxygen, sulfur, and nitrogen atoms of fenticonazole and the PPARγ residues Tyr327, Cys285, and Ser342 [98].

**Figure 27:**
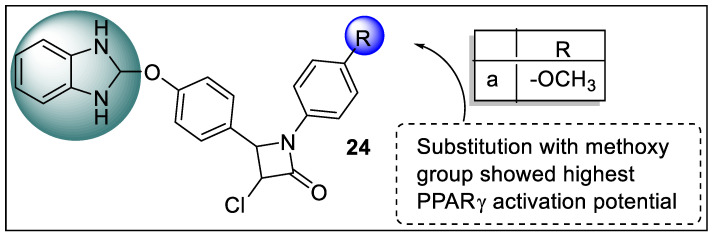
Structural activity relationship of Benzimidazole hybrids (**24**) as potent PPAR ligands.

Recently, Bhalla et al. carried out a synthesis of a new series of C4-benzimidazolyloxyphenyl-substituted trans-β-lactam analogs by changing various aliphatic and aromatic functionalities on imine and ketene viz. –Cl, –SC_6_H_5_, –SeC_6_H_5_, –OCH_3_, –C_6_H_4_.Cl(p), –C_6_H_4_.OCH_3_(p) and –CH_2_C_6_H_5_. The synthesized analogs were screened for molecular docking with PPAR using FLEXx software to determine their antidiabetic potential. When compared to the C3 functionality of the β-lactam unit, the docking analysis demonstrated that N1 functionality has a significant impact on the binding affinity of the molecules. Furthermore, β-lactam **24a** with a paramethoxyphenyl group at N1 shows promise for future anti-diabetic drug development (Figure 27) [99].

### 5.4. Thiazole

**Figure 28:**
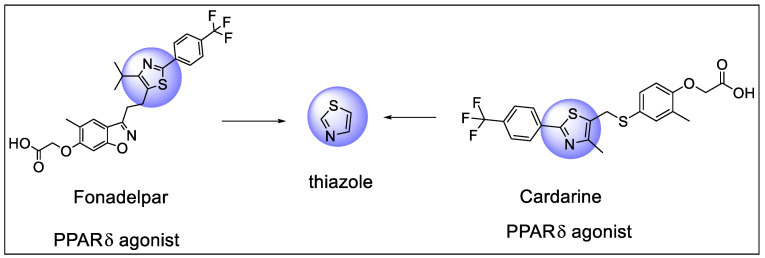
Thiazole scaffold containing PPAR ligands present in the market.

Thiazole is a five-membered heterocyclic molecule with one sulfur and one pyridine-type nitrogen atom at position three of the cyclic ring structure. The thiazole core, found in the center unit of molecules, exhibits PPAR activity as well as other biological functions (Figure 28).

**Figure 29:**
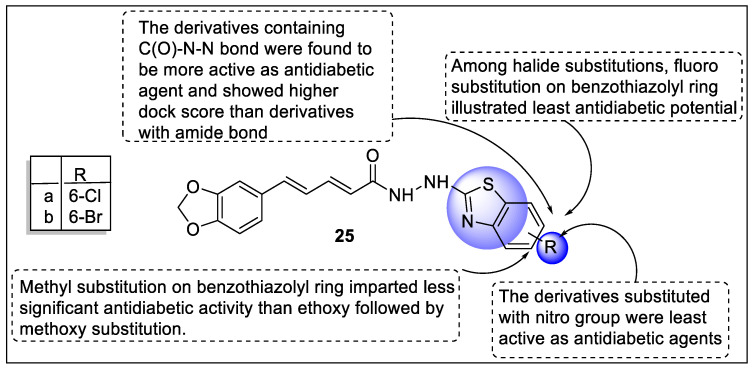
Structural activity relationship of thiazole hybrids (**25**) as potent PPAR ligands.

Piperine is a dietary *alkaloid* responsible for the pungency of black pepper (Family: *Piperaceae*) so Kharbanda et al. carried out the isolation of Piperine from *Piper nigrum L.* after hydrolysis and treated it under basic conditions to produce piperic acid, which served as a precursor in the synthesis of piperine analogs that contains benzothiazole moiety. The antidiabetic potential of all piperine-containing benzothiazole derivatives was screened using the OGT test, followed by an assessment of active derivatives using the STZ-induced diabetic model. Interestingly, synthesized piperine analogs exhibited promising anti-diabetic action as compared to rosiglitazone (standard). Furthermore, the agonistic effect of these active analogs towards PPARγ was tested, demonstrating their mode of action. Most of the synthesized piperine derivatives were active antidiabetic agents with compounds **25a** and **25b** showing maximum effect (Figure 29). These compounds showed their activity by increasing PPARγ gene expression. Additionally, compounds **25a** and **25b** did not promote excess body weight, which is normally observed in patients suffering from diabetes [100].

**Figure 30:**
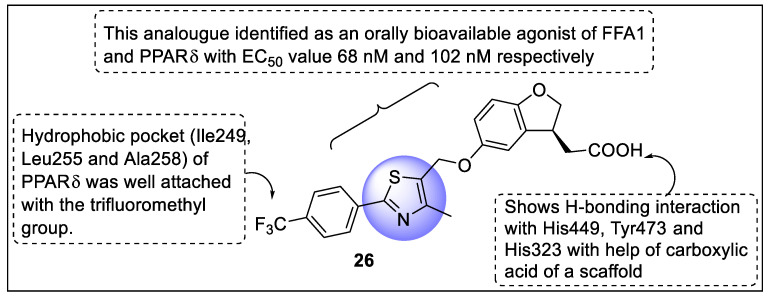
Structural activity relationship of thiazole hybrids (**26**) as potent PPAR ligands.

Li et al. designed some hybrid compounds of AM-4668, CP-1 (FFA1 agonist), and GW501516 (selective PPARδ agonist) as dual agonists of FFA1 and PPARδ in the treatment of several metabolic disorders. The potential of hybrid analogs was screened on the FFA1 and PPARδ receptors using Chinese hamster ovary cells and Gal4 receptor cell-based assay, respectively. Analog **26** was identified as an orally bioavailable agonist of FFA1 and PPARδ with EC_50_ values of 68 nM and 102 nM, respectively, which also showed selectivity toward PPARs. Along with selectivity and potency, analog **26** displayed a good pharmacokinetic profile with good plasma exposure and decreased glucose level. The docking studies revealed that analog **26** showed H-bonding interaction with His449, Tyr473, and His323 with the help of carboxylic acid as a scaffold. Moreover, the hydrophobic pocket (Ile249, Leu255, and Ala258) of PPARδ was well attached to the trifluoromethyl group of left benzene (Figure 30) [101].

**Figure 31:**
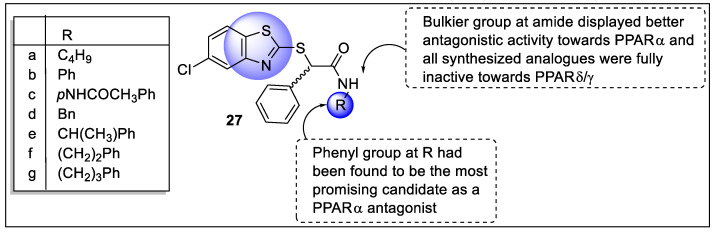
Structural activity relationship of thiazole hybrids (**27**) as potent PPAR ligands.

Ammazzalorso *et al.* designed a series of benzothiazole derivatives to antagonise the activation of PPARs. The activity of the synthesized analogs was investigated on the human PPARs through transactivation assay using Wy-14,643, L-165,041, and rosiglitazone as references for PPARα-δ-γ, respectively. The study revealed that analogs **27a**–**g** exhibited inactivity for activation of PPARα-δ-γ. Thus, researchers investigated the antagonist potential of analogs via a competitive binding assay that resulted in analogs **27a**–**g** selectively antagonized PPARα with a 22–57% range of inhibition. Analogs **27d**–**g** containing bulkier group at amide displayed better antagonistic activity toward PPARα and all synthesized analogs were comparatively inactive towards PPARδ-γ (Figure 31). The anti-proliferative action of synthesized analogs was explored using various tumor cells such as AsPC-1 and Capan-2 for pancreatic, HT-29 and SW480 for colorectal, along with PTJ64i and PTJ86i for paraganglioma cancer cell lines. With regard to referencing PPARα agonist (GW6471), analog **27b** reduced the PTJ64i and PTJ86i cell viability (inhibition rate >90%) and also exhibited viability in pancreatic and colorectal cancer cells (up to 68–75%). Therefore, analog **27b** was the most promising candidate as a PPARα antagonist [102].

**Figure 32:**
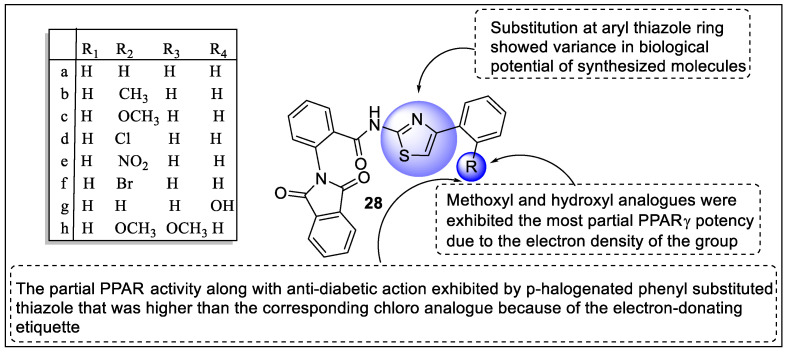
Structural activity relationship of Thiazole hybrids (**28**) as potent PPAR ligands.

Enhancing insulin sensitivity via targeting PPARγ and β-catenin is one of the attractive pathways for the treatment of T2DM; therefore, Mourad et al. synthesized analogs of α-phthalimido-o-toluoyl2-aminothiazole hybrids and carried out the anti-diabetic activity. Three of the synthesized analogs modulated PPARγ gene expression that displayed partial agonistic activity towards PPARγ. Upon treatment of compounds with 3T3-L1, the cells displayed induction PPARγ2 mRNA level. Moreover, compounds **28c, 28g,** and **28a** showed better PPARγ2 mRNA levels compared to the reference drug. Therefore, **28c, 28g,** and **28a** also displayed 4–5 fold more PPARγ activity than rosiglitazone. The structural activity relationship studies revealed that substitution at the aryl thiazole ring showed variance in the biological potential of synthesized molecules. Accordingly, methoxyl (**28c**) and hydroxyl (**28g**) analogs exhibited partial PPARγ potency due to the electron density of the group (Figure 32). Intriguingly, the partial PPARγ activity along with anti-diabetic action exhibited by p-halogenated phenyl-substituted thiazole **28f** was higher than the corresponding chloro analog (**28d**). The docking study revealed that synthesized analogs had distinctive attachments at the LBD of PPARγ and generated H-bonding interaction with Ser342 amino acid residue [103].

**Figure 33:**
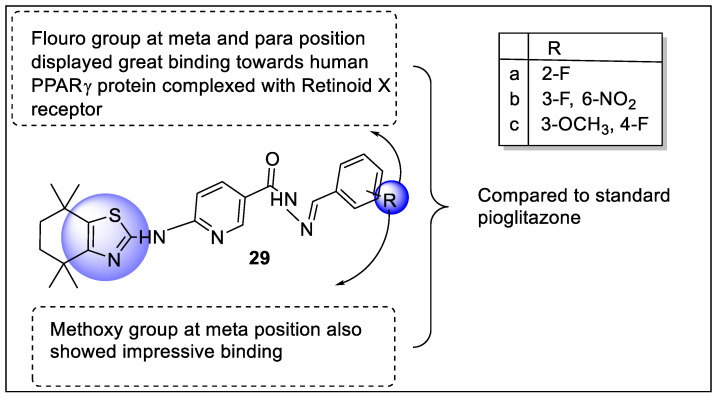
Structural activity relationship of thiazole hybrids (**29**) as potent PPAR ligands.

Recently, Patchipala et al. reported some new analogs of the thiazole-pyridine scaffold by condensation of 4,4,7,7-tetra-methyl-4,5,6,7-tetrahydrobenzo[d]thiazol-2-amine with 6-chloronicotinate and evaluated for their anti-diabetic activity by in vivo housing Swiss albino mice. The in silico screening of these analogs was performed with human PPAR-γ protein complexed with RXR alpha Nuclear Receptor (PDB ID: 3DZY). All synthesized compounds showed a reduction in glucose level when compared with glibenclamide where fluoro and nitro (**29b**) containing analog had the highest activity. Additionally, the in silico binding studies revealed that fluorobenzaldehyde with methoxy (**29b**) and nitro (**29c**) displayed impressive interaction with human PPAR-γ complexed with RXR alpha Nuclear Receptor in comparison to Rosiglitazone (Figure 33) [104].

### 5.5. Indole

**Figure 34:**
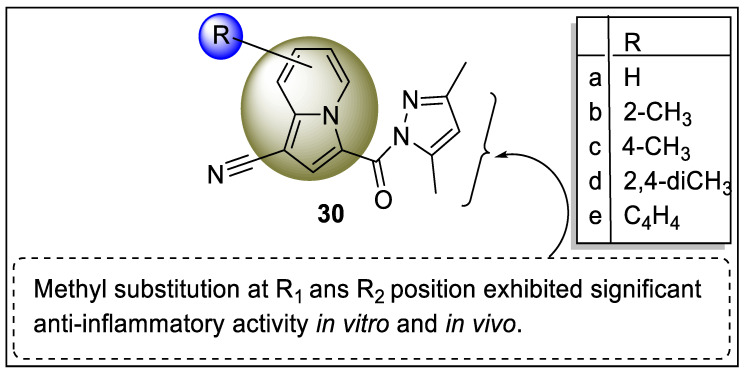
Structural activity relationship of indol hybrids (**30**) as potent PPAR ligands.

The aromatic heterocyclic organic compound indole is a six-membered benzene ring fused to a five-membered pyrrole ring, giving it a bicyclic structure. In 2017, Fu et al. reported a new series of pyrazole-containing indolizine (**30a**–**e**) and screened for anti-inflammatory activities for the treatment of Crohn’s disease. The pharmacological evaluation performed using LPS-stimulated peritoneal macrophages revealed that analog **30d** displayed a reduction in secretion of TNF-α which was the outcome of the activation of the macrophages. Interestingly, the western blotting, Luciferase assay, and immunofluorescence showed positive results regarding the potency of analog **30d** and also helped in the mechanism of anti-mucosal inflammatory potential. The mechanism underlying that, analog **30d,** activates the PPARγ receptor that leads to suppression of NK-κB activation (60 µm), and the result was similar to the 5-ASA (5-Aminosalicylic acid) (Figure 34) [105].

**Figure 35:**
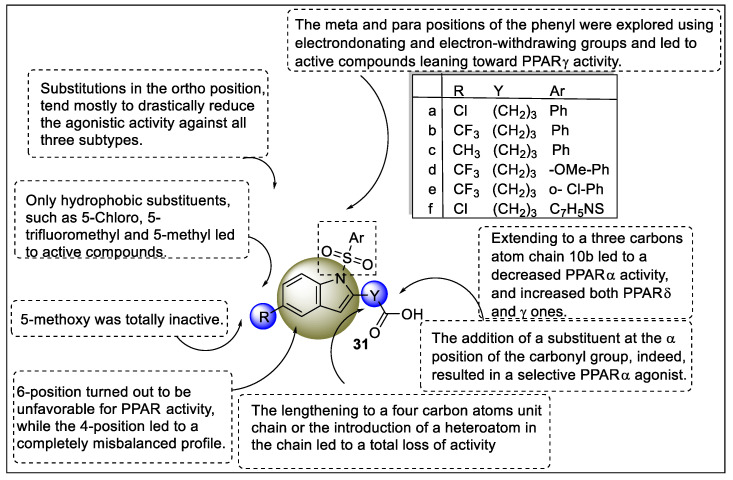
Structural activity relationship of indol hybrids (**31**) as potent PPAR ligands.

The following year, Boubia et al. reported a series of indol sulphonamide analogs to find suitable candidates for the activation of PPARα/δ/γ. Moreover, compounds were tested for human PPARs in vitro study utilizing cell-based Gal-4 transactivation assays. The structures of the proposed analogs contain three pharmacophoric features such as indole, chain linker, and aryl sulphonamide. The SAR study revealed that chain linker with a three-carbon atom chain exhibited an optimum PPARα/δ/γ profile at potency and efficacy level. (**31a**, Emax = 50%). Therefore, substitutions at the fifth position of indole were screened and mainly hydrophobic groups such as 5-Chloro, 5-trifluoromethyl, and 5-methyl showed good activity towards PAN PPARs, whereas 6-position and 4-position were shown to be adverse and fully unbalanced for PPAR activity. Interestingly, using electron-donating and electron-withdrawing groups, the para and meta locations of the phenyl of aryl sulphonamide resulted in active compounds that inclined toward PPARγ activity (Figure 35). Finally, lanifibranor showed strong anti-fibrotic action in mice using the liver fibrosis model, as well as outstanding anti-hyperglycemic and hypolipidemic potential [106].

**Figure 36:**
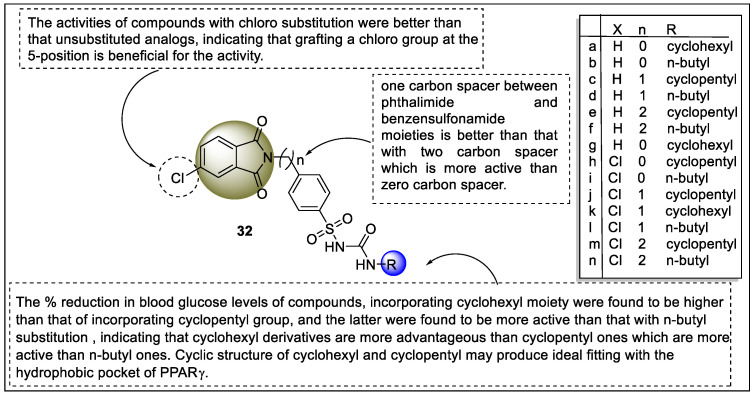
Structural activity relationship of indole hybrids (**32**) as potent PPAR ligands.

El-Zahabi and co-workers reported some analogs of phthalimide-sulfonylurea hybrids that act as PPARγ ligands. The biological evaluation of hybrid analogs was carried out through in vivo anti-hyperglycaemic activity in streptozotocin-induced hyperglycaemic rats using glibenclamide (sulfonylurea drug) and an oral glucose tolerance test (OGTT) that showed compounds **32c**, **32d, 32g, 32h, 32j,** and **32k** to exhibit a potential 24.43 to 21.43% reduction in blood glucose level in comparison to Glibenclamide (29.3%). However, the activity was less than the reference drug. On the other hand, the result of the OGTT revealed promising PPARγ agonistic activity via increasing glucose utilization and insulin sensitization. In silico studies were also carried out using four different methods including molecular docking, pharmacophore screening, QSAR, and ADMET. The docking studies were performed on a PPARγ crystallographic structure (PDB ID: 1FM6) using MOE software. Impressive free energies against PPARγ were found in compounds **32c, 32d, 32j**, and **32m**. When compared to the generated pharmacophore model, compounds **32c**, **32j**, **32k**, **32l**, and **32n** displayed the best match values (Figure 36). The QSAR studies highlighted that these compounds possessed significant binding affinities towards PPARγ and insulin secretion [107].

### 5.6. Furan

Furan is a heterocyclic organic molecule made up of a five-membered aromatic ring containing four carbon atoms and one oxygen atom. Various furan derivatives as PPAR ligands have been thoroughly investigated in terms of chemistry and biological activity over the years.

**Figure 37:**
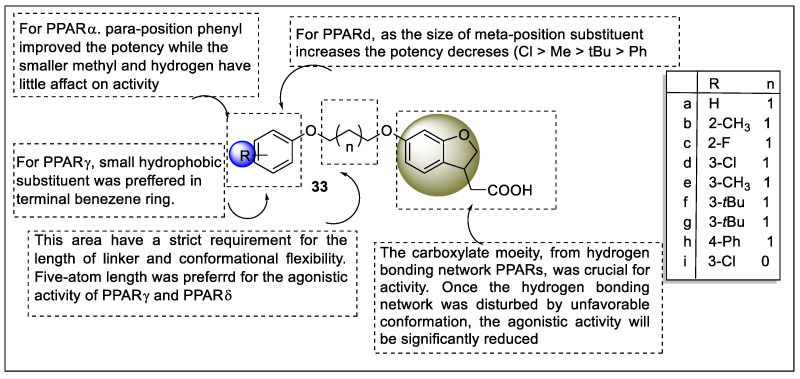
Structural activity relationship of Furan hybrids (**33**) as potent PPAR ligands.

Li et al. reported a series of hybrid compounds of PPARs and FFA1 agonists to elucidate the dual agonistic activity for the treatment of type 2 diabetes. The PPAR agonistic activity of these hybrid compounds was tested by Gal4 receptor cell-based assay using GW7647, Rosiglitazone, and GW0742 as the positive control. Hybrid compound **33a** increased the PPARγ agonistic activity and led to a 20-fold decrease in PPARδ agonistic activity. The length of the linker also affected the potency, wherein shortening the linker decreased the potency of PPARγ and PPARδ. Compounds **33b** and **33c,** which are 2-position substitution derivatives, exhibited impressive PPARγ and PPARδ agonistic activity compared with the parent compound **33a**. Various substitutions were carried out at 3-position for FFA1 and PPARs. The agonistic activity of 3-Cl (**33d**) > 3-Me (**33e**) >3-tBu (**33f**) > 3-Ph (**33a**) for PPAR suggested that the steric effect at the 3-position may affect the strength of the receptor (Figure 37). Due to the small ligand-binding pocket at this location, PPAR has a low affinity for ligands. Especially, the 4-position phenyl-substituted derivative **33h** showed greater agonistic activity on the PPARα in comparison to other analogs. Induced-fit docking studies were also performed on the FFA1, PPARγ, and PPARδ by using PDB codes: 4PHU, 2Q8S, and 1GWX, respectively. The resulting compound **33i** showed multiple types of interaction with amino acid residues. Hydrogen-bonding interactions were displayed by the acidic moiety and carboxylic acid of compound **33i** with Tyr473, His449, Ser289, Tyr473, Tyr327, Arg2258, and Arg183 [108].

**Figure 38:**
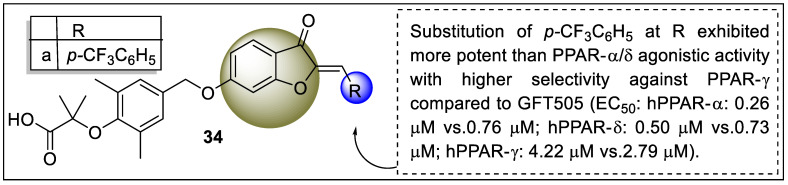
Structural activity relationship of Furan hybrids (**34**) as potent PPAR ligands.

Recently, Liu et al. synthesized a series of hybrid compounds as dual agonists of PPAR-α/δ. The rationale behind the synthesis was a hybridization of the carboxylic group of GFT505 with the parent motif of sulfuration to generate a low-risk and more potent PPAR ligand. These synthesized candidates were tested for the agonistic activity of human PPARα, δ, and γ by in vitro GAL4-PPAR transactivation assay on transfected COS7 cells whereas GW7647, GW501516, and rosiglitazone were used as reference. The results of the in vitro assay found that compound **34a** displayed 16.2-fold and 8.4-fold more PPAR-α/δ agonistic activity than PPARγ and also exhibited better agonistic activity (Figure 38). Molecular docking was also performed in Schrödinger 2009 using PDB ID: 4CI4 (PPARα) and 3SP9 (PPARδ). Compound **34a** and GFT505 were docked, wherein compound **34a** displayed hydrogen bonding with Tyr314, His 440, Ser 280, and Tyr464 residues of PPARα and His 287, Thr252, Thr253, His413, and Tyr437 residues of PPARδ [109].

**Figure 39:**
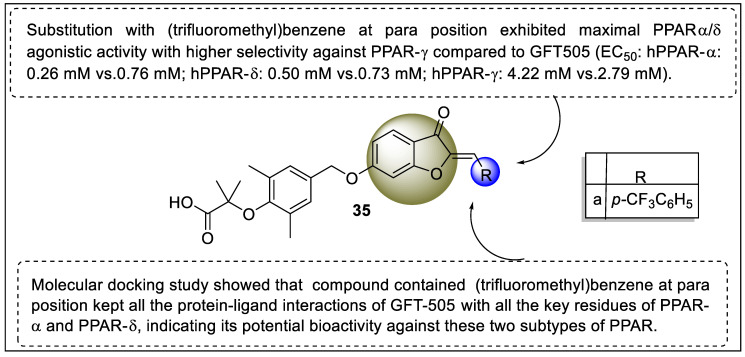
Structural activity relationship of Furan hybrids (**35**) as potent PPAR ligands.

Liu et al. synthesized a series of novel dual PPAR-α/δ agonists to target PPARs. The in vitro evaluation revealed that the compound substituted with trifluoro carbon possessed high potency toward PPAR- α/δ (0.26 ± 0.08 µM 0.50 ± 0.10 µM) and higher selectivity against PPARγ (−4.22 ± 0.18 µM) than that of GFT505 (Figure 39). The molecular docking studies also showed considerable binding affinity towards LBD of PPARα/δ compared to GFT505 that displayed H-bond interactions between the ligand and important amino acids such as Tyr314, His 440, Ser 280, and Tyr464. Moreover, H-bond interactions were displayed between the ligand and His 287, His 413, Thr252, Thr437, and Tyr253 of PPAR δ [110].

### 5.7. Benzopyran

Benzopyrans are polycyclic molecules in which a benzene ring and pyran ring are fused with various levels of saturation. The name benzopyran is widely used to refer to polycycles fused with a pyran ring (chromenes) but also applies to heterocycles bearing a dihydropyran (chromans). A lot of benzopyrans have been explored for their potential for PPARs.

**Figure 40:**
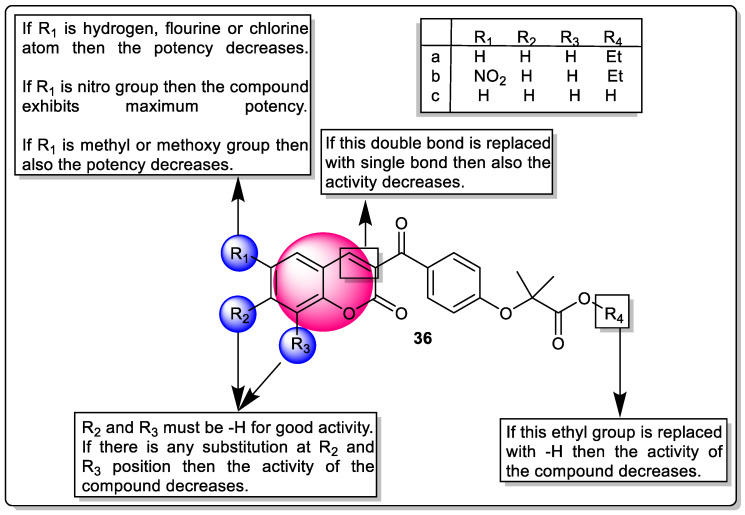
Structural activity relationship of coumarin–chalcone hybrids (**36**) as potent PPAR ligands.

Niu et al. reported a series of coumarin–chalcone fibrates that exert an agonistic effect on PPARα/γ along with antioxidant potential. The pharmacological evaluation of the synthesized molecule was carried out through transactivation assay using firefly luciferase reporter gene technology in HERK293 cells. Fenofibrate (PPARα) and rosiglitazone (PPARγ) were used as positive control. Among these synthesized analogs **36a**, **36b** (most potent), and **36c** exhibited significant agonistic potential towards PPARα with EC_50 −_12.69 µM, −6.99 µM (4.3-fold more potent than Fenofibrate), and −11.03 µM, respectively. In addition, analogs, **36a**, **36b,** and **36c** also displayed PPARγ agonistic activities with EC_50_ -5.19 µM, −0.91 µM, and −11.03 µM, respectively. The SAR study showed that alteration at C6′ position with a nitro group (electron-withdrawing in nature) raises the agonistic efficacy towards PPARα/γ (Figure 40). Moreover, benzopyran moiety with a double bond was required for the PPARα/γ potential [111].

**Figure 41:**
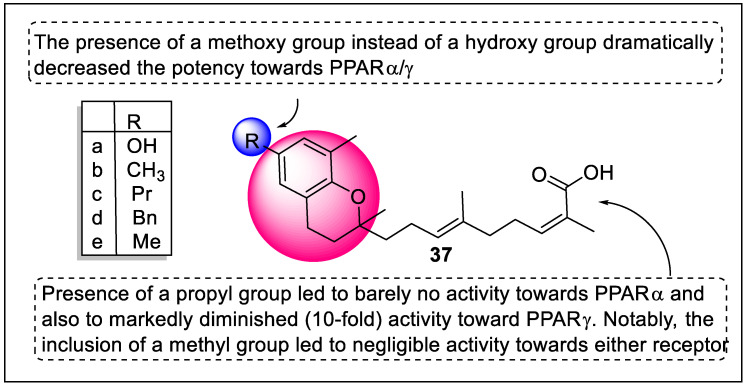
Structural activity relationship of benzopyran hybrids (**37**) as potent PPAR ligands.

Bermejo and their team reported prenylated benzopyrans polycerasoidol (**37a**) and polycerasoidin (**37b**) and their semisynthetic analogs as potential PPARα/γ agonists. These nine analogs were screened for PPARα/δ/γ activities using a transactivation assay that revealed polycerasoidol and its analog (**37c**) exhibited agonistic activity towards PPARγ and the % value was 95% and 86%, respectively. Interestingly, analog **37d** displayed maximum activity towards hPPARα and γ with % values of 191% and 88%, respectively (Figure 41). The importance of these groups for optimum ligand binding to the PPARα (PDB code: 4BcR) and PPARγ (PDB code: 4eMA) domains was discovered by molecular modeling. His323, Tyr327, His449, and Tyr473 form significant H-bond interactions with Polycerasoidol. At the C-9′ position of 1, the carboxylic acid group was found to be the preferred PPARγ receptor binding site and served as an anchoring point in docking research and MD simulations. Furthermore, polycerasoidol (**37a**) inhibited mononuclear leukocyte adherence to the defective endothelium in a concentration-dependent manner through RXR/PPARγ interactions, resulting in a powerful anti-inflammatory impact [112].

**Figure 42:**
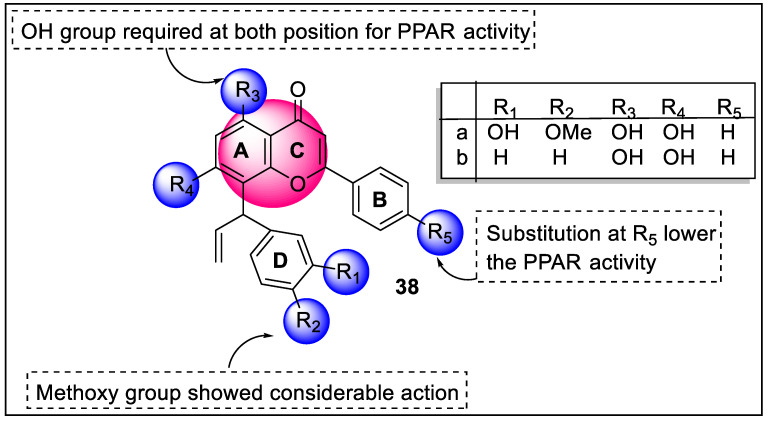
Structural activity relationship of chromenone hybrids (**38**) as potent PPAR ligands.

5,7-dihydroxy-8-(1-(4-hydroxy-3-methoxyphenyl)allyl)-2-phenyl-4*H*-chromen-4-one (**38**) was found to be a lead compound for adiponectin secretion-inducing activity isolated from the natural honeybee propolis. Ahn et al. designed and synthesized **38** and its analogs along with the screening of activity towards PPARs. According to the target identification investigation, analogs **38a** and **38b** interacted directly with PPARα/δ/γ. Overexpression of HO-1 mediated by compound **38a** is most likely linked to PPAR pan-modulatory potential, accepting the fact that PPAR agonists can kill cancerous cells. The binding processes of chromenone analogs showed similarity with PPARα/γ partial agonists; according to a docking study between **38b** and the PPAR-LBD, they connect with the hydrophobic LBD of PPARs. Analog **38b** did not affect WY14643 or pioglitazone’s ability to induce adiponectin secretion. Compound **38b** may have a different effect on adiponectin secretion than a specific PPAR partial agonist (Figure 42). Furthermore, **38b** demonstrated distinct cellular phenotypic consequences in the transcriptional control of lipid metabolic enzymes, separating it from selective PPAR mono-agonists [113].

**Figure 43:**
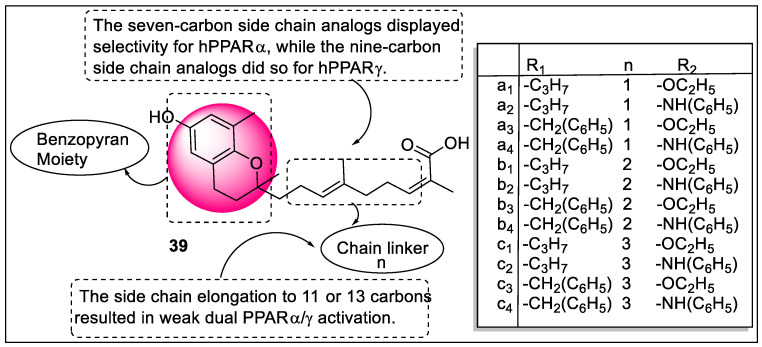
Structural activity relationship of benzopyran hybrids (**39**) as potent PPAR ligands.

2-Prenylated benzopyrans (PB) address a category of natural and synthetic agents displaying a broad scope of potential. Polycerasoidol is a characteristic PB found in *Polyalthia cerasoides* (*Annonaceae*) from their stem bark that shows both PPARα and PPARγ agonistic potential. Recently, in 2022, Vila et al. designed three series of prenylated benzopyrans containing multiple motifs such as polycerasoidol and trans-δ-tocotrienolic acid along with isoprenoid units in the hydrocarbon side chain at the 2-position of the chroman-6-ol motif and carried out their hPPAR transactivation activity. The pharmacological evaluation revealed that the seven-carbon side-chain analogs (a_1_–a_4_) showed selectivity for hPPARα, while the nine-carbon side-chain analogs (b_1_–b_4_) did the same for hPPARγ. The side chain extension to 11 or 13 carbons (c_1_–c_4_) brought about weak activation of PPARα/γ. Hence, 2-prenylated benzopyrans of the seven- and nine-carbon side chain (polycerasoidol analogs) are great lead compounds for creating valuable contenders to forestall cardiovascular illnesses related to metabolic disorders (Figure 43) [114].

### 5.8. Bavachinin (BVC)

**Figure 44:**
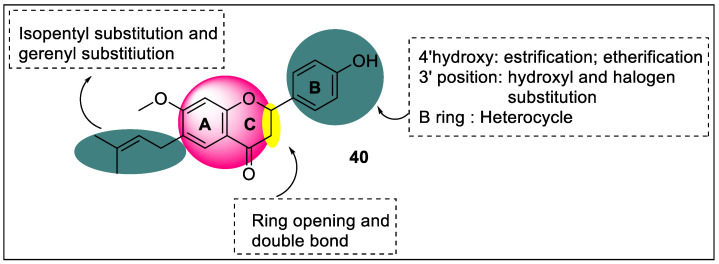
Series of bavachinin hybrids (**40**) as potent PPAR ligands.

Bavachinin (7-O-Methylbavachin) is a novel natural pan-PPAR agonist from the fruit of the traditional Chinese glucose-lowering herb malaytea scurfpea. It shows stronger activities with PPAR-γ than with PPAR-α and PPAR-β/δ (EC_50_  = −0.74 μmol/L,−4.00 μmol/L, and −8.07 μmol/L in 293T cells, respectively). Many derivatives have been synthesized from these natural compounds. Du et al. carried out the synthesis of a similar type of modification in bavachinin derivatives for PPARγ agonist candidates. In total, 30 molecules, including flavanone and flavone analogs, were tested by reporter gene assays for PPARγ agonistic potency. From the pharmacological results, the SAR study revealed that when an isopentenyl group and methoxy group attached at the C-6 and C-7 positions, respectively, they showed better PPARγ agonist activity in the A-ring. At the C-6 position, the findings also imply that an aliphatic chain is necessary for PPAR agonistic potential. Altering the hydroxyl group at C-4’ to an aromatic ester group improves PPARγ agonistic potential, but changing the hydroxyl group to an aliphatic ester or ether lowers it in the B-ring. The phenyl ring structure is required for PPARγ agonistic potential, and substituting thiazole, imidazole, or pyridine rings reduces it significantly. Alteration of a single bond at C-2 and C-3 position to double bond improved the PPARγ agonist activity in the ring-C (Figure 44) [115].

**Figure 45:**
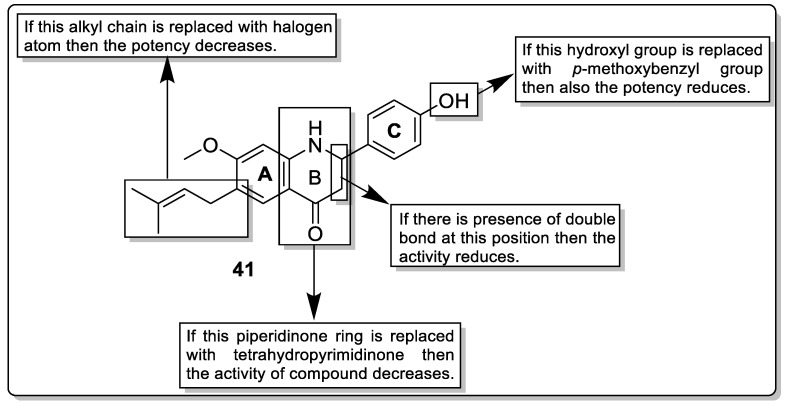
Structural activity relationship of bavachinin hybrids (**41**) as potent PPAR ligands.

Yi et al. designed and synthesized derivatives of bavachinin with variation at ring A, ring B, and ring C and tested for pan-PPAR agonistic activity using reporter gene assays. The order in which the N element appears is important for the pan-PPAR agonist activity; N-substituted analogs at the C3 and C1, 3 positions (of the C-ring) lowered pan-PPAR agonistic potential, and C1 position N-substitute derivative **41** increased the activity. The findings imply that adding N-atom to the C1 did not affect BVC’s pan-PPAR agonistic action (Figure 45). When compared to bavachinin, Analog **41** is a powerful pan-PPAR agonist with significantly higher PPAR α/β agonistic potential and similar PPAR-γ agonistic potential. [116].

### 5.9. Miscellaneous

**Figure 46:**
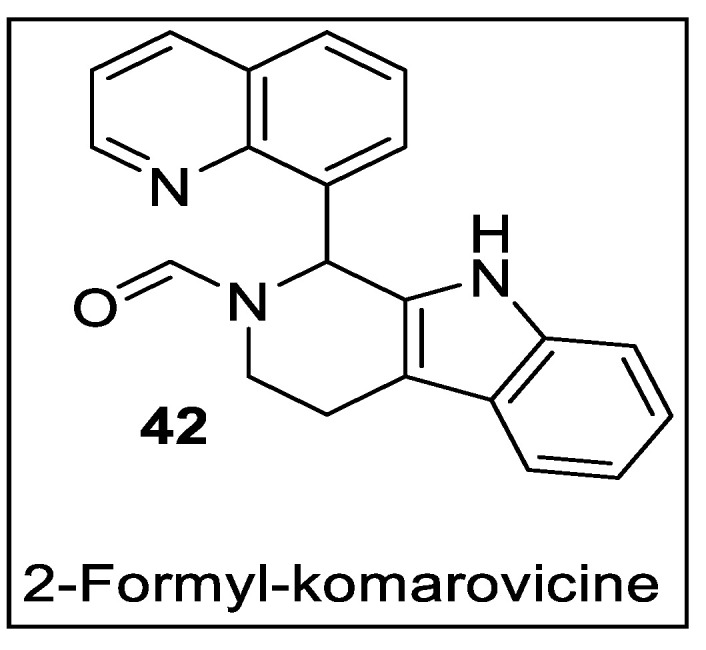
Chemical structure of 2-formyl-komarovicine (**42**).

Adiponectin is a key adipocytokine produced by adipocytes in mammals. Low adiponectin expression has been linked to a variety of human metabolic disorders and malignancies, Ahn et al. synthesized 2-formyl-komarovicine (Figure 46) and evaluated adiponectin secretion by performing the phenotypic assay on human bone marrow–mesenchymal stem cells (hBM-MSCs). The synthetic 2-formyl-komarovicine induced the production of adiponectin and displayed binding towards PPARγ in a concentration-dependent manner. The molecular docking studies revealed that 2-formyl-komarovicine connected with the PPARγ ligand-binding domain’s hydrophobic pocket, but had an inability to interact and stabilize helix H12 [117].

**Figure 47:**
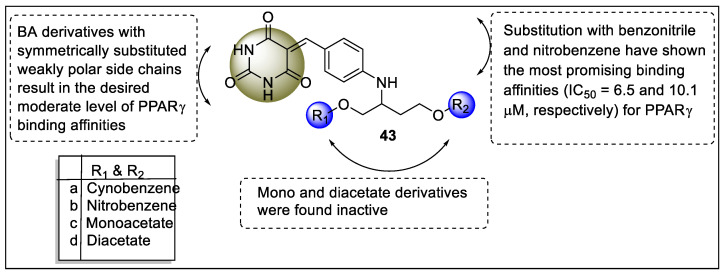
Structural activity relationship of barbituric acid (BA) hybrids (**43**) as potent PPAR ligands.

Dixit et al. designed Y-shaped barbituric acid (BA) derivatives via molecular docking and then synthesized fourteen of them using the OH-protection-deprotection technique for PPARγ activation. The time-resolved FRET technique was used for the analysis of the competitive binding of synthesized molecules towards PPARγ. The binding affinity of symmetrically substituted derivatives was higher than that of unsymmetrically substituted derivatives. The binding affinities of nitrobenzyl (**43b**) and cyanophenyl (**43a**)**-**substituted derivatives were found to have better activity with −10.1 µM and −6.5 µM, respectively, while monoacetate (**43c**) and diacetate (**43d**) derivatives were devoid of activity (Figure 47).

The in silico studies revealed that synthesized analogs showed H-binding interaction with the H-3 helix (amino acid residues = Ser289, His449, His323) and the H-12 helix (Tyr473). When synthesized analogs were substituted with the hydrophobic group, they displayed a score similar to the rosiglitazone. The most important keynote was BA derivatives along with substitution with weakly polar side chains achieve the requisite good level of PPARγ binding affinities [118].

**Figure 48:**
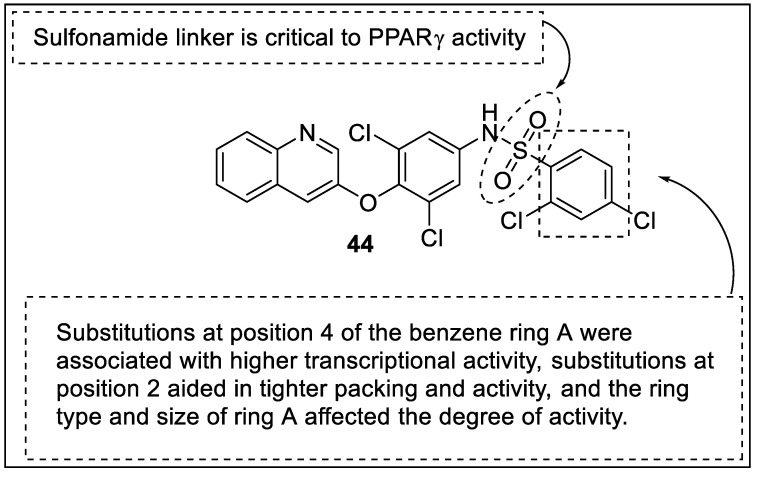
Structural activity relationship of INT131 hybrids (**44**) as potent PPAR ligands.

2,4-dichloro-N-(3,5-dichloro-4-(quinoline-3-yloxy)phenyl)benzenesulfonamide (INT131) had great potential toward PPARγ receptors, with a Ki of 10 nM; therefore, Frkic et al. designed and synthesized analogs of the INT131 utilizing chemical alteration of the A ring. This SAR investigation determined the impact of A ring substituents, their positions on the A ring, and various rings at position A on transcriptional activity, as well as how these substituents affect the compound **44** scaffold’s interaction with the PPARγ receptor. Seven ligands with improved PPARγ potency were discovered in the SAR of compound **44** analogs. They differed from **44** in that they had bulky replacements at position 2 of benzene A, which facilitate better lock-and-key fitting in the PPARγ LBD. The SAR data further highlighted the relevance of a sulfonamide linker in position A, as well as a substituted, 6-membered benzyl ring (Figure 48) [119].

**Figure 49:**
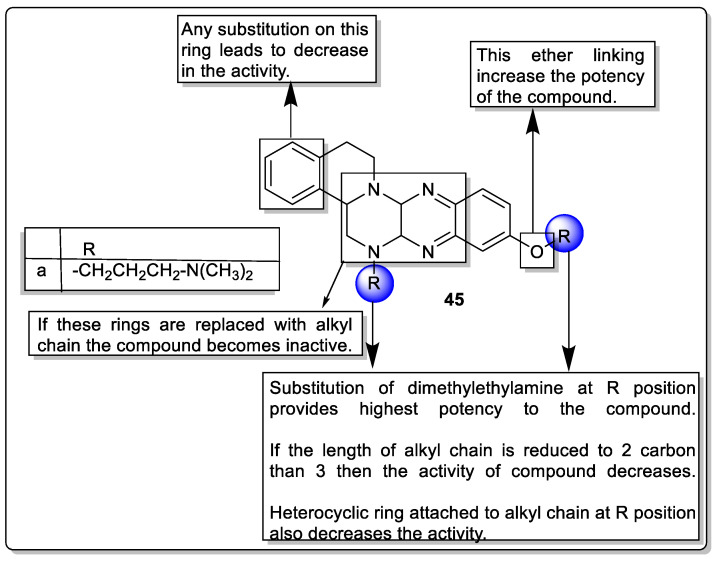
Structural activity relationship of isoquinoline hybrids (**45**) as potent PPAR ligands.

Tetrazanbigen is a sterol isoquinoline analog that exhibits potential toward human cancer cell lines through induction of lipoapoptosis, but this compound has some disadvantages such as poor water solubility and low efficiency. Therefore, Gan et al. synthesized a series of Tetrazanbigen analogs intending to improve water solubility and anti-cancer activities via acting on PPARγ. The CCK-8 assay revealed that analog **45a** showed promising inhibition of HepG2 and A549 cell growth with IC_50_ values of -0.54 and -0.47 μM, respectively. Furthermore, the in vitro antiproliferative activity was performed by using an in vivo xenograft model in which analog **45a** exhibited a reduction of tumor growth at a dose of 10 mg/kg. Moreover, the water solubility of the analog **45a** was found to be 31.4 mg/mL, which was 1000-fold higher than that of Tetrazanbigen (4 μg/mL) (Figure 49) [120].

**Figure 50:**
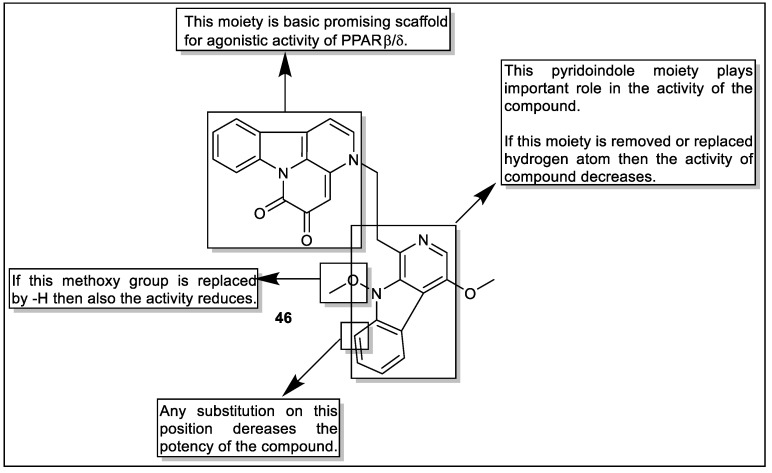
Structural activity relationship of picrasidine N hybrids (**46**) as potent PPAR ligands.

Using a mammalian one-hybrid experiment, Zhao et al. extracted and found the naturally occurring picrasidine N from *Picrasma quassioides* (family: *Simaroubaceac*) as an agonist of PPARβ/δ and described from a library of plant extracts. The biological evaluation proved that compound **46** exhibited activity towards PPARβ/δ. Furthermore, analog **46** increased PPARβ/δ transcriptional activity in a PPRE-driven luciferase reporter gene test. Furthermore, analog **46** preferentially increased ANGPTL4 mRNA expression. This is in contrast to those known as PPARβ/δ agonists (Figure 50), which have been shown to stimulate the expression of not only ANGPTL4 but also additional PPARβ/δ target genes such as PDK4, ADRP, and CPT-1 [121].

**Figure 51:**
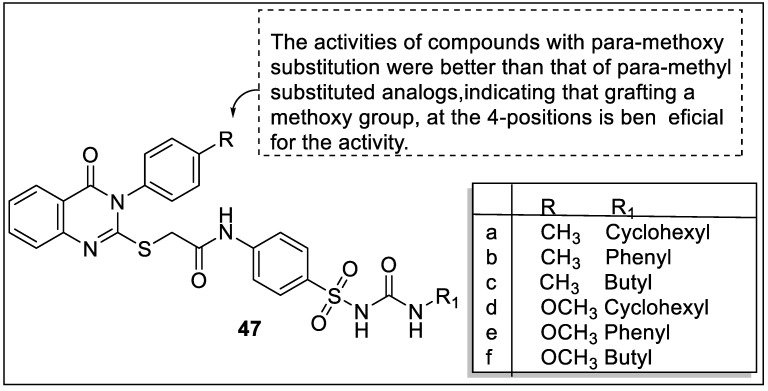
Structural activity relationship of quinazoline hybrids (**47**) as potent PPAR ligands.

**Figure 52:**
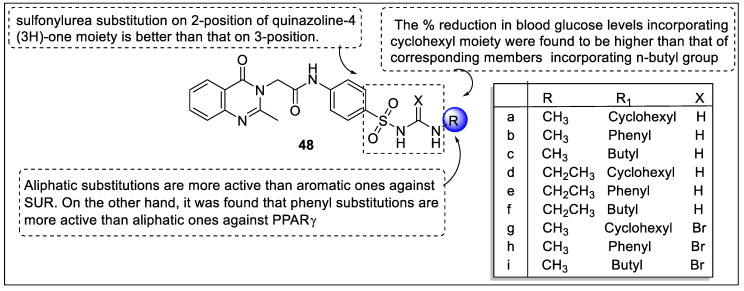
Structural activity relationship of quinazoline hybrids (**48**) as potent PPAR ligands.

Ibrahim et al. designed quinazoline-4(3*H*)-one sulfonylurea hybrids that exert dual agonistic activity of PPARγ and SUR. The biological evaluation of synthesized derivatives was screened for in vivo anti-hyperglycemic activity in streptozotocin-induced hyperglycemic rats where glibenclamide and rosiglitazone were used as a reference drug. In vitro PPARγ-ligand binding and insulin assay were also examined. Based on the biological evaluation, compound **47d** reduced the 46.42% blood glucose level and become the most potent candidate. Beside compound **47a, 47c** (Figure 51), **48a, 48h** and **48r** (Figure 52) reduced 33.42%, 37.76%, 38.64%, 34.25% and 38.44%, respectively. The result of the PPARγ binding affinity test using fluorescence polarization assay found that compounds **47b, 47d, 47f, 48f,** and **48g** possessed the highest affinities with IC_50_ values -0.371, -0.350, -0.369, -0.408, and -0.353 µM, respectively. The most potent insulin-secreting activities were exhibited by compounds **47d**, **47f**, and **48d** with EC_50_ values of -0.97, -1.01, and -1.15 µM, respectively. Docking and pharmacophore studies revealed that compounds **47d, 48e, 48f,** and **48g** had significant dock scores and PPARγ binding affinity. Compounds **47a, 47c, 47f, 48d**, and **48i** had the best binding values to the pharmacophore model [122].

**Figure 53:**
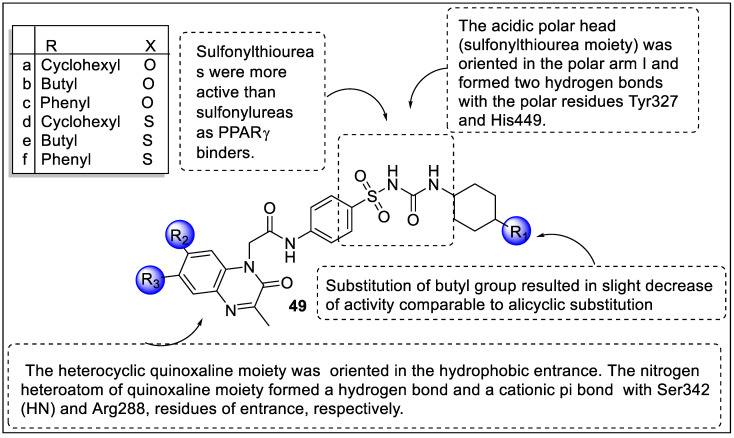
Structural activity relationship of quinoxaline hybrids (**49**) as potent PPAR ligands.

The same group of researchers reported quinoxaline derivatives exhibiting dual agonistic activity of PPARγ and SUR. Based on the biological evaluation, compound **49a** reduced the 50.58% blood glucose level and became the most potent candidate. Besides, compound **49e** was reduced by 43.84%. The results of the PPARγ binding affinity test using Fluorescence Polarization Assay revealed that compounds **49a**, **49b**, **49d,** and **49e** showed higher affinities with IC_50_ values of -0.482, -0.491, -0.350, and -0.369 µM, respectively. The most potent insulin-secreting activities were exhibited by **49a**, and **49b** with EC_50_ values of -0.92 and -0.98 µM, respectively. Docking tests were carried out along with pharmacophore investigations to support the biological activity of the synthesized compounds against SURs (Figure 53). The results of molecular modeling studies revealed that the majority of the produced compounds had a significant affinity for PPAR and SUR [132].

**Figure 54:**
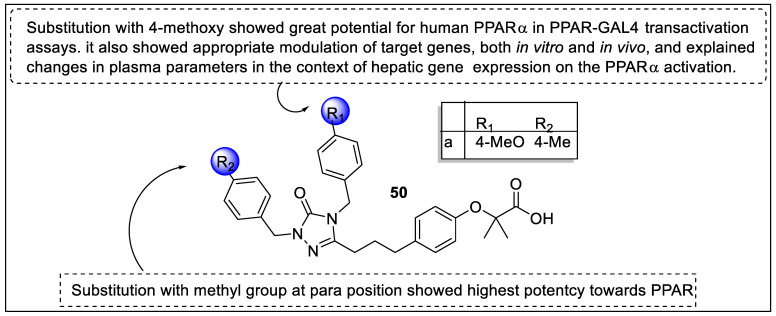
Structural activity relationship of triazole hybrids (**50**) as potent PPAR ligands.

LY518674 is a potent agonist of PPARα that has already progressed to clinical trial phases, so based on the pharmacophore of LY518674, Yu et al. reported a novel DY series of PPARα modulators for NAFLD treatment. The pharmacological evaluation of synthesized analogs revealed that analogs exhibited promising activity at the PPARα. Furthermore, the analogs were inactive towards PPARα and PPARδ at up to 10–30 µm and displayed selectivity towards PPARα (potency EC_50_ = -0.85 – -12 nM). The docking study also supported the results of the pharmacological evaluation and displayed major interaction of **50a** with PPARα (PDB id 1k7l), such as 4-methoxybenzil tail at R1 attached with lipophilic region B, and showed hydrophobic interaction with the amino acid residues (I272/V332/I339/L344). At R_2_, the site c region showed π-stacking interaction of 4-methyl benzyl moiety with F273 and F351 at a distance of 6.3 Å between ring centers (Figure 54) [123].

**Figure 55:**
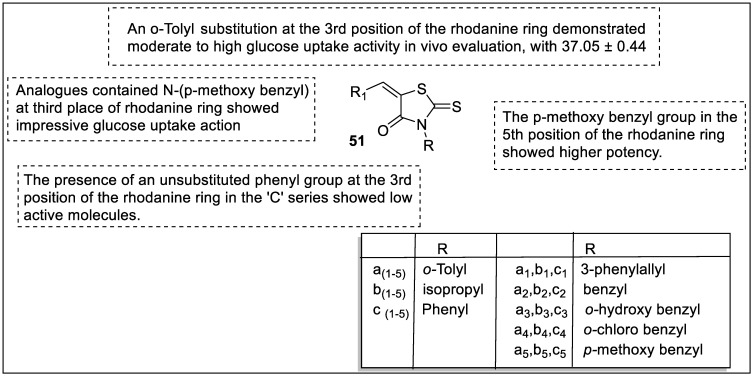
Structural activity relationship of rhodanine hybrids (**51**) as potent PPAR ligands.

Mandal et al. designed analogs of rhodanine with phenyl replacement at the fifth place of the ring to find new ligands for the PPAR-γ receptor for conceivable antihyperglycemic potential and carried out their molecular modeling against PPAR-γ (PDB ID: 2PRG). All analogs were tested for in vitro glucose uptake assay. The analogs containing *p*-methoxy benzyl (**51a_5_** and **51b_5_**) ring showed impressive glucose uptake action. Therefore, both of the analogs were screened for the in vivo antihyperglycemic study which resulted in a more impressive antihyperglycemic potential than pioglitazone. The SAR studies revealed that compounds with an o-Tolyl (**51a_1_**_–**5**_) at the third position demonstrated moderate to high glucose uptake activity in vivo evaluation, with 37.05 ± 0.44 being the most active value compared to pioglitazone (39.18 ± 0.63). The isopropyl (**51b_1–5_**) at the third position in the ‘B’ family of compounds demonstrated moderate activity. The presence of an unsubstituted phenyl group at the third position of the rhodanine ring in the ‘C’ series showed low active molecules, probably due to the lack of strong intermolecular interaction revealed by docking analysis. The *p*-methoxy benzyl group in the fifth position of the rhodanine ring showed higher potency due to its favorable interaction with protein amino acids, as evidenced by molecular docking, implying that an electron-donating group at the para position of the benzyl substitution may increase activity, whereas an electron negative group at the ortho position of the benzyl substitution may result in moderate activity (Figure 55). Thus, the rhodanine ring is required for glucose absorption activity; nevertheless, electron-donating substitutions at the third and fifth positions of the rhodanine nucleus may yield a contender [124].

**Figure 56:**
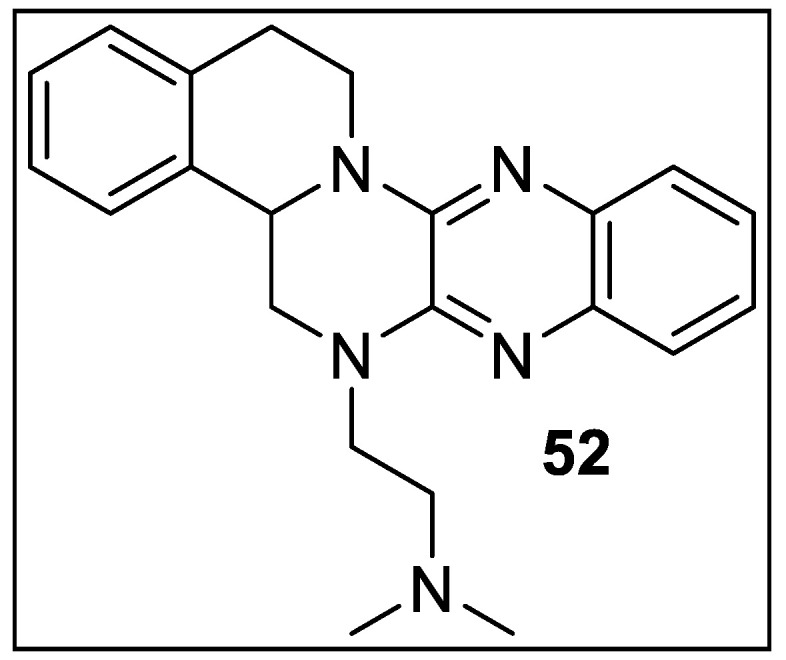
Chemical structure of TNBG-5602.

TNBG-5602 (**52**) is a recently designed compound containing an isoquinoline motif. Thus, Hu et al. showed the anticancer impact of **52** using in vitro and in vivo models and examined its conceivable anticancer potential. The antiproliferation impact of **52** in vitro was assessed in human liver disease cell line QGY-7701. The antiproliferation action of **52** in vivo was surveyed in a xenograft model. The outcome of the CCK-8 test revealed that **52** can successfully restrain the expansion of liver disease cells in vitro. In a xenograft liver disease model, **52** could astoundingly hinder the development of growths. During in vitro and in vivo examinations, the scientist noticed that **52** initiated the lipid collection in malignant growth cells and tissues. Moreover, the review demonstrated that the anticancer impact of **52** might be applied through enacting PPARγ and downregulating multiplying cell atomic antigens (PCNA). They found that **52** can expand the outflow of PPARγ at the mRNA and protein levels in vitro and in vivo. Further investigation discovered that a PPARγ agonist (RSG) can upgrade the lipid aggregation of **52**, while a PPAR_λ_ inhibitor (T0070907) can switch the lipid-amassing of **52**. Also, RSG can significantly improve the antiproliferation of **52**, while T0070907 can to some extent turn around the antiproliferation impact of **52** in QGY-7701 cells. Moreover, RSG can improve the downregulation of PCNA of **52**, while T0070907 can invert the downregulation of PCNA of 52 (Figure 56). This information recommended that **52** instigated lipid accumulation and antiproliferation in liver disease cells by expanding the declaration of PPARγ to diminish the expression of PCNA [125].

**Figure 57:**
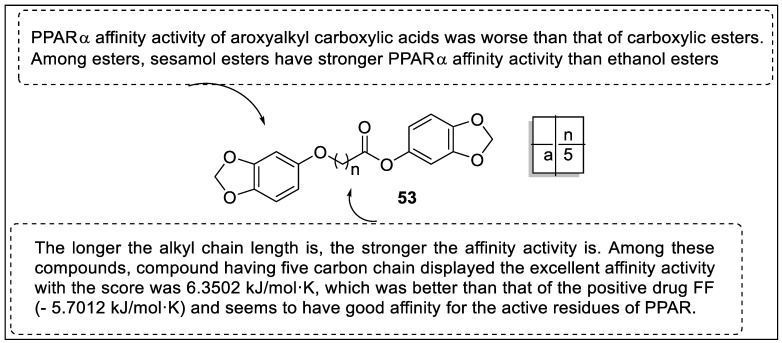
Structural activity relationship of 1,3-benzodioxole hybrids as potent PPAR ligands.

Xie et al. designed 1,3-benzodioxole-based fibrate scaffold analogs and screened them using hyperlipidemia mice. The pharmacological evaluation revealed that analogs with longer alkyl chain length showed more impressive activity against hyperlipidemia than fenofibrate (FF). They also exhibited a promising reduction of plasma lipids, such as total cholesterol (TC), triglycerides (TG), and low-density lipoprotein cholesterin (LDL-C). The affinity activity of the protein PPAR (PDB ID: 1K7L) was studied using the docking approach. The PPARα binding activity of aroxyalkyl carboxylic acids was shown to be worse than that of carboxylic esters in these molecular docking investigations. Sesamol esters have a higher PPARα affinity than ethanol esters among esters. Sesamol has a synergistic role, and its PPARα affinity activity was greatly increased following esterification with carboxylic acid, despite its low PPARα affinity activity. They also discovered that the affinity of these analogs for PPARα was related to the length of the alkyl chain (n) between the aroxy and carbonyl groups. Affinity increases with the length of the alkyl chain (Figure 57). Compound **53a** had the best affinity activity, with a score of -6.3502 kJ/molK, higher than the FF (-5.7012 kJ/molK), and appears to have an impressive affinity for the PPARα. In the Triton WR 1339 hyperlipidemic mice model, the results of docking were similar to the results of pharmacological evaluation [126].

**Figure 58:**
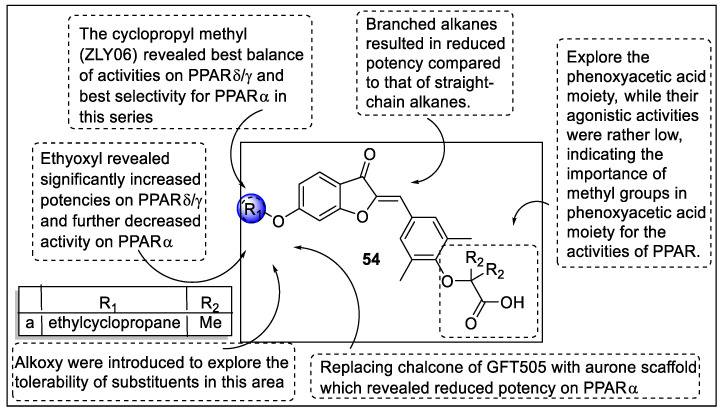
Structural activity relationship of GFT505 hybrids as potent PPAR ligands.

Li et al. identified a partial agonist of dual PPARα/δ by using a conformational restriction strategy. The discovery of a PPARα/δ partial agonist (ZLY06 (**54a**)) was the resulting structure of cyclization of GFT505. The SAR studies displayed that the chalcone part of the GFT505 replaced with aurone scaffold reduced the PPARα potency but induced potency towards PPARγ. Ethyoxyl compound exhibited potency towards PPARδ/γ but reduced the PPARα activity. Herein, the alkoxy series were selected for further substitution that revealed straight-chain alkanes had more active than branched alkanes. Therefore, ZLY06 displayed best activities towards PPARδ/γ and selectivity towards PPARα. Interestingly, phenoxy acetic acid-containing analogs had low agonistic activity towards PPARs but displayed the contribution of methyl and phenoxy acetic acid moiety (Figure 58). The pharmacological evaluation of ZLY06 revealed a significant reduction in glucose levels in HF/STZ and *ob/ob* mice. It also promoted glucolipid metabolism without gaining weight, fatty acid oxidation, and inhibition of hepatic lipid accumulation [127].

## 6. Conclusions

The involvement of PPARs with multiple pathophysiological conditions has been categorically documented in the last two decades. Scientists have attempted to target these receptors with a variety of heterocyclic compounds in a bid to come up with novel medicaments for disease conditions. This article has successfully compiled the published reports on the use of various heterocycles over the last few years (2016–2022) where significant advancements have been made towards the development of PPAR ligands as potential medication focuses with a promising therapeutic profile. An attempt has been made to briefly portray PPARs receptors, their physiological role, and market drug use, along with clinical information. Further, the fusion of different heterocyclic cores to bring about improvement in terms of their efficacy and interactions has also been highlighted. In addition to this, current advancements in the field of the medicinal chemistry of PPAR ligands, with the latest reference to structural alteration on heterocyclic cores, as well as the mechanism of action, SAR, and in silico analysis, have also been discussed. Overall, this article should provide an excellent commentary for prospective researchers in the field.

## 7. Future Perspectives

PPARs are still being investigated in terms of their potential in metabolic diseases, despite the controversy surrounding the clinical activities of PPAR agonists. It is important to note that those SAR investigations demonstrated that PPAR ligands play a role in T2DM, cancer, inflammation, and/or non-alcoholic fatty liver disease in a largely consistent manner. Numerous investigations using mouse models and human surrogate studies have been demonstrated. Each PPAR agonist must be judged on its own merits because of the variety of SAR actions and toxicity. As mentioned in this review, the activation of the PPAR family of genes is complex and unexpected. It may be the balance of stimulation of α, β, γ, or individuals that determines efficacy and toxicity, including late toxicity. There is no “class effect” within the PPAR family; therefore, each agent needs to be viewed as an individual. Each agent in this class must undergo an individual evaluation, including clinical outcome studies, as required by the FDA. The activation or inhibition of PPARs may have a variety of negative impacts since they regulate a wide range of cell processes in different organs. Indeed, with different PPAR agonists, significant therapeutic, as well as toxicological profile variations, have been noted, posing difficult problems for the field’s researchers. The area is primed for a renewed surge in interest and effort to develop novel PPAR-centered therapeutics for numerous diseases with the arrival of the relatively recent discoveries of PPAR receptor allostery and allosteric ligands. These discoveries are anticipated to result in the creation of a new class of PPAR modulators called allosteric ligands, which will have less toxicity and more focused pharmacological effects than canonical ligands.

## Figures and Tables

**Table 1:** Various patents on Peroxisome proliferator-activated receptor.

Sr No	Date	Patent Number	Description	Ref.
1.	19 August 2021	WO2021161218	Sulfinic acid and sulfonic acid compounds for use in modulating peroxisome proliferator-activated receptors	[73]
2.	23 December 2020	US20210238125	Diabetes and Metabolic Syndrome Treatment with a Novel Dual Modulator of Soluble Epoxide Hydrolase and Peroxisome Proliferator-Activated Receptors	[74]
3.	18 July 2018	US20190077774A1	Peroxisome proliferator-activated receptor agonists	[75]
4.	28 July 2018	US20180305403A1	PPAR agonists and methods of use thereof	[76]
5.	01 July 2018	US20190000790A1	PPAR-γ activators and their therapeutical usage	[77]
6.	13 December 2017	US20190060269A1	Brain-Derived PPARα Ligands	[78]
7.	07 April 2017	US20180015062A1	PPAR-α Activator, Pharmaceutical Composition, Food and Drink, Food Additive, Supplement, and Method of Manufacturing the Same	[79]
8.	13 April 2017	WO2017180818A1	PPAR agonists, compounds, pharmaceutical compositions, and methods of use thereof	[80]
9.	07 April 2017	US20170304255A1	PPAR agonists, compounds, pharmaceutical compositions, and methods of use thereof	[81]
10.	30 January 2017	US20170210711A1	Competitive PPAR-α antagonists	[82]

**Table 2:** Summary of various heterocycles with the different substitutions for Peroxisome proliferator-activated receptor.

Heterocycles	Most Potent Substitution	Activity	Reference
Thiazolidinediones	Allyl derivative (**1a**)	EC_50_ of −4.95 μM in the human PPAR-γ transactivation assay	[83]
	Amino derivative (**3b**)	1.7 times more than reference compounds in glucose uptake assay	[84]
	Chloro derivative (**8a**)	63.15%, PPAR-γ transactivation	[85]
	Dichloro derivative (**9a**)	(−11.6930) Docking score	[86]
	Nitro and carboxylic acid derivative (**10d**)	(−17.44) Docking score	[87]
	Flouro derivative (**12e**)	Reduced 0.09-fold the expression gene of PPARγ in cultured adipocytes compared to the control group	[88]
Oxadiazole	No substitution (**13d**)	EC_50_ = −0.15 for PPARα EC_50_ = −0.29 for PPARδ	[89]
	ADAM (**14**)	2.5-fold greater efficacy in activating PPARα	[90]
	Bromo derivative (**15a**)	Potency and selectivity towards PPARα/δ receptors with PPARα/δ/γ EC_50_, EC_50_ γ/α ratio, and EC_50_ γ/δ ratio value was 8/5/2939 nM, 367, 588 respectively	[91]
	Flouro derivative (**16d**)	PPARα −0.06 ± 0.0005, PPARγ −0.07 ± 0.0006	[92]
Bemzoimidazole	Chloro derivative	EC_50_ = −0.19 ± 0.01	[93]
	Flouro and chloro derivative	−68 ± 28^j^	[94]
	(**20c**)	−10.6 ± 0.4	[95]
	Chloro and iodo derivative	Ki = 1023 µM for PPARγ and Ki = 0.106 µM for PPARδ	[96]
	Chloro derivative (**22b**)	PPARα 307 PPARγ 2052 PPARδ 214	[97]
	(**23**)	Kd value = −2.8 ± 0.8 nM	[98]
	Methoxy derivative (**24a**)		[99]
Thiazole	-	-	[100]
	(**26**)	EC_50_ > 10 µM	[101]
	Phenyl derivative (**27b**)	PPARα Imax% 22 ± 16	[102]
	p-halogenated phenyl substituted thiazole derivative (**28f**)	Increased PPARγ activity by almost 4- to 5-fold while rosiglitazone exhibited approximately 10-fold activation	[103]
	fluoro and nitro derivative (**29b**)	-	[104]
indole	2,4 dimethoxy derivative **30d**	-	[105]
	Chloro and phenyl derivative (**31a**)	PPARα/δ/γ profile at potency and efficacy level.(31a, Emax = 50%)	[106]
	Cyclohexyl derivative	24.43% Reduction in blood glucose level	[107]
Furan	phenyl derivative **33h**	hPPARα (LBD)-GAL4 EC_50_ (µM) −7.31 hPPARγ (LBD)-GAL4 EC_50_ (µM) −2.97 hPPARδ (LBD)-GAL4 EC_50_ (µM) −1.98	[108]
	Triflouro carbon phenyl derivative (**34a**)	16.2-fold and 8.4-fold more PPAR-α/δ agonistic activity than PPARγ	[109]
	Triflouro carbon phenyl derivative (**35a**)	High potency toward PPAR- α/δ (0.26 ± 0.08 µM 0.50 ± 0.10 µM) and higher selectivity against PPARγ (4.22 ± 0.18 M) than that of GFT505	[110]
Benzopyran	Nitro derivative (**36b**)	EC_50_ −0.91 µM	[111]
	Butane derivative (**37c**)	hPPARα and γ with % values of 91% and 88% using a transactivation assay	[112]
	Di-hydroxyl deritive (**38b**)	Ki value 1.41 μM	[113]
	**39**	hPPARα with high selectivity (123 % and 38% for α and γ, respectively).	[114]
Bavachinin	(**40**)	PPARα agonistic activity EC_50_ −0.43	[115]
	**41**	21 (EC_50_ = −22.28 μM)	[116]
Miscellaneous	**42**	-	[117]
	Cyanophenyl (**43a**)	IC_50_ 6.5 µM time-resolved FRET technique	[118]
	**44**	EC_50_ (nM) = −170 ± 10	[119]
	**45a**	HepG2 and A549 cell growth with IC_50_ values of −0.54 and −0.47 μM, respectively, CCK-8 assay	[120]
	**46**	-	[121]
	Methoxy and cyclohexyl derivative	IC_50_ values −0.350 fluorescence polarization assay	[122]
	**50a**		[123]
	o-Tolyl (**51a_1–5_**)	glucose uptake activity in vivo evaluation, with −37.05 ± 0.44	[124]
	**52**	-	[125]
	**53a**	-	[126]
	Ethylcyclopropane derivative (**54a**)	-	[127]

## List of Abbreviations

5-ASA	5-Aminosalicylic acid
ACCs	Acetyl-CoA carboxylase
ADAM	6-(5-heptyl-1,2-oxazol-3-yl)hexanoic acid
BVC	Bavachinin
CPT1	Carnitine palmityl transferase 1
FF	Fenofibrate
hBM-MSCs	human bone marrow-mesenchymal stem cells
LBP	Ligand-binding pocket
LDL-C	Low-density lipoprotein cholesterin
MDS	Molecular design suite
NHR assay	Nuclear hormone assay
OFAI	Oxohexadecenoic acids (7*E*)-9-oxohexadec-7-enoic
OFAII	(10E)-9 -oxohexadec-10-enoic acid
OGTT	Oral glucose tolerance test
PB	2-Prenylated benzopyrans
PPAR	Peroxisome proliferator-activated receptors
PPREs	Peroxisome proliferator hormone response elements
RXR	Retinoid X receptor
SAR	Structural activity relationship
T2DM	Type 2 diabetes mellitus
TC	Total cholesterol
TG	Triglycerides TR-FRET: the time-resolved fluorescence resonance energy transfer
TZD	Thiazolidinedione
